# Promoting mental well-being among secondary school students in Vietnam using the Y-MIND app: A protocol for a hybrid type 2, sequence pre-post, quasi-experimental study

**DOI:** 10.1371/journal.pone.0332875

**Published:** 2025-10-07

**Authors:** Jill K. Murphy, Vu Cong Nguyen, Dang Thuy Linh, Hui Xie, Thu H. Tran, Skye P. Barbic, Leena W. Chau, Hasina Samji, Harry Minas, Minh Vu Nguyen, Anthony Obrzut, John O’Neil, Erin E. Michalak, Raymond W. Lam

**Affiliations:** 1 Interdisciplinary Health Program, St. Francis Xavier University, Antigonish, Nova Scotia, Canada; 2 Institute of Population, Health and Development, Hanoi, Vietnam; 3 Faculty of Health Sciences, Simon Fraser University, Burnaby, British Columbia, Canada; 4 Department of Psychiatry, University of British Columbia, Vancouver, British Columbia, Canada; 5 Department of Occupational Science and Occupational Therapy, University of British Columbia, Vancouver, British Columbia, Canada; 6 Foundry, BC, Vancouver, British Columbia, Canada; 7 Global and Cultural Mental Health Unit, Centre for Mental Health, Melbourne School of Population and Global Health, University of Melbourne, Melbourne, Australia; PLOS: Public Library of Science, UNITED KINGDOM OF GREAT BRITAIN AND NORTHERN IRELAND

## Abstract

Youth mental health is recognized as an urgent priority worldwide. Life skills education and self-management skills delivered at the population level are evidence-based approaches to promote mental well-being and resilience among youth and digital technologies are identified as promising approaches for delivering these interventions. The study objective is to assess implementation and clinical outcomes of the delivery of a life skills and self-management intervention delivered at the population level via an app (Y-MIND) in Vietnamese secondary schools. Y-MIND was co-designed with Vietnamese youth to promote appropriateness, acceptability and uptake. We will conduct a hybrid type 2, sequence pre-post, quasi-experimental study in twelve schools across three Vietnamese provinces. Participants will be students aged fifteen years who provide assent to participate and receive parental consent. Control and intervention cohorts from participating schools will be enrolled in subsequent years. The primary clinical outcome measure is well-being measured using the Warwick Edinburgh Well-Being Scale. Secondary outcomes will explore factors including resilience, common mental health conditions, positive childhood experiences, academic stress, internet addiction, substance use and experiences of bullying and punishment. Implementation outcomes include app engagement, uptake, retention and qualitative interviews to understand acceptability and appropriateness of the intervention. Assessments will be administered via an online survey using Qualtrics at baseline, six and twelve months. The intervention cohort will receive access to the app, while the control cohort will receive usual care. The control cohort will have access to the app following the study period, but no app-use data will be collected. Generalized Linear Mixed-Effect Models, with the school specified as the clustering variable, will be used for all outcomes data. Understanding the potential for clinical effectiveness and successful implementation of the Y-MIND app will contribute important evidence to the fields of global youth mental health, digital health and implementation science.

**Trial registration:**

This study is registered at ClinicalTrials.gov (NCT06753344).

## Introduction

### Background and rationale

Youth (generally defined as young people between the ages of 15–24 years [1]) worldwide experience substantial mental health challenges, with depression and anxiety among the leading causes of morbidity and suicide as the third leading cause of mortality among this age group [2]. Despite this, mental health promotion, prevention, and support services for youth are severely limited, especially in low and middle-income countries (LMICs). Vietnam has a population of 97 million people, 70% of whom are under the age of 35 years [3]. Like in many LMICs, Vietnam has limited mental health promotion, prevention, early intervention, and care availability for youth [4].

Life skills-based programs, delivered at a population-level via schools, are an evidence-based approach that have the potential to improve mental health, resilience and well-being among youth in low resource settings. The integration of life skills into schools has been recommended by UNICEF to support youth mental well-being in Vietnam [5]. Globally, though schools are recognized as promising environments to deliver mental health promotion programs, limited capacity in school settings (e.g., human resources, curricular time) can act as a barrier [6]. In Vietnam, school health staff, who provide basic medical care (e.g., first aid) and health education, are assigned to every school, with qualifications and roles varying across the country. School health staff often have limited training, lack access to consistent continuing professional development and have a limited level of knowledge about mental health and mental health promotion in schools [7]. A 2021 systematic review found that more robust evidence, including implementation research, is needed to support the implementation and scale-up of appropriate life skills-based interventions in LMICs [8]. Digital health approaches including interventions delivered via mobile apps are also increasingly recognized as an effective, acceptable and accessible approach to promoting youth mental health and well-being. However, more research on digital health approaches is needed, particularly within LMICs [9–12].

Though large-scale epidemiological mental health research is limited in Vietnam, the results of a nationally representative household survey on adolescent mental health conducted in 2021 found that 21.7% of Vietnamese adolescents had experienced a mental health problem in the last 12 months [13]. Evidence further suggests that common mental disorders and psychosocial stress are a considerable challenge among youth in school. In Ho Chi Minh City, high school students showed high levels of stress (36.1%), depression (39.8%) and anxiety (59.8%) [14] while among high school students in Can Tho, 23% reported anxiety, 41.1% reported depression symptoms and 18.7% had symptom levels indicative of Major Depressive Disorder (MDD) [15]. A 2020 cross sectional study among students aged 13–17 years in four Vietnamese provinces across diverse geographical regions found that 31.7% had depressive symptoms and 11% reported experiencing suicidal ideation in the last year [16].

Though there is limited nationally representative epidemiological data about risk factors for poor mental health among youth in Vietnam, several studies identify potential risk factors. Excessive workload at school, high levels of academic pressure and feeling disengaged from school or teachers are identified in several studies as risk factors for poor mental health and well-being among students in Vietnam [5,14,15,17,18]. Other risk factors range from female gender [5,9,15,16], struggles with sexual identity or identifying as a sexual or gender minority [5,17,19,20], social or emotional isolation [5,21], experiences of bullying [16,17] conflict with teachers [15], poor communication with parents or caregivers [5], and low socioeconomic status [22]. Alcohol use was identified as a risk factor for depressive symptoms and findings from the 2019 Youth Risk Behaviour Survey show that among high school students in Hanoi over half reported alcohol use [17]. Internet addiction is also a concern among Vietnamese youth and results from a 2017 cross-sectional survey among 566 adolescents show it is associated with higher prevalence of depression and anxiety and with poor quality of life outcomes [23]. The COVID-19 pandemic exacerbated risk factors for youth in Vietnam, with the enduring effects of social isolation and increased Internet use during the pandemic identified as detrimental to mental well-being [13,24]. In a 2020 study among students aged 13–17 years [16], those in the older age ranges had a higher prevalence of depression symptoms, leading authors to recommend targeting mental health and wellbeing interventions among older adolescents.

Vietnamese youth also have low mental health literacy and are unsure of where to obtain support, preferring to seek help from informal sources such as friends, family, online and on social media [14]. The 2021 Vietnamese Adolescent Mental Health Survey [13] found that only 8.4% of youth with a mental health problem had access to care. In a study on depression and suicidality among 661 secondary school youth in Hanoi, 31.2% reported that they had never been informed about the prevention and treatment of depression and suicidality. 43.1% of these students said they could not easily access care, with the most common barriers being the lack of available services (38.9%) and cost (23.9%) [17]. Poor mental health during adolescence is associated with many social and quality of life risk factors including poor school performance, high-risk behaviour, substance misuse, and with challenges later in life including lower socioeconomic status and negative physical and mental health outcomes [25,26]. Conversely, building positive mental health, resilience and coping skills at this critical life stage has positive impacts that may resonate throughout the lifespan [27]. This underscores the critical need for an accessible, population-level intervention to promote well-being and prevent mental ill health among youth in Vietnam.

Most of the research on mental health, well-being and resilience among youth comes from high-income countries, with more research from LMICs needed [12,28]. The use of digital mental health interventions like smart phone apps has previously shown promise in Vietnam but there has been a lack of engagement with end users in their development and design that has contributed to low engagement [29]. The Youth Promotion of Resilience Involving Mental E-Health (Y-PRIME) study responds to the gap in the availability of evidence-based interventions to promote mental health, well-being and resilience for youth in Vietnam by assessing the implementation and clinical outcomes of an app-based life skills and self-management intervention (Y-MIND). Y-MIND was co-designed with Vietnamese youth and will be evaluated in a study using a hybrid type 2 [30], quasi-experimental, sequenced pre-post implementation design. We hypothesize that an app-based intervention, co-designed with Vietnamese youth, will lead to positive implementation outcomes among youth and within the implementation environment. We also posit that it will lead to improved well-being among Vietnamese youth.

## Materials and methods

### Overall study design and objectives

Y-PRIME is a five-year study taking place in two phases. In Phase 1, we worked with a Vietnamese Youth Advisory Committee (V-YAC) made up of thirteen Vietnamese youth aged 15–24 years from across the country. We collaborated with the V-YAC to co-design an app-based intervention (Y-MIND) that will be tested among 15-year-old Vietnamese secondary school students in Phase 2. The co-design process consisted of focus groups with V-YAC members, a two-day app-development workshop using persona development methods [31], and an iterative process involving regular meetings and discussions between the study team, V-YAC members and a local Vietnamese software development company (OneDay Software) over several months. Details of the co-design process will be reported elsewhere.

**Fig 1:**
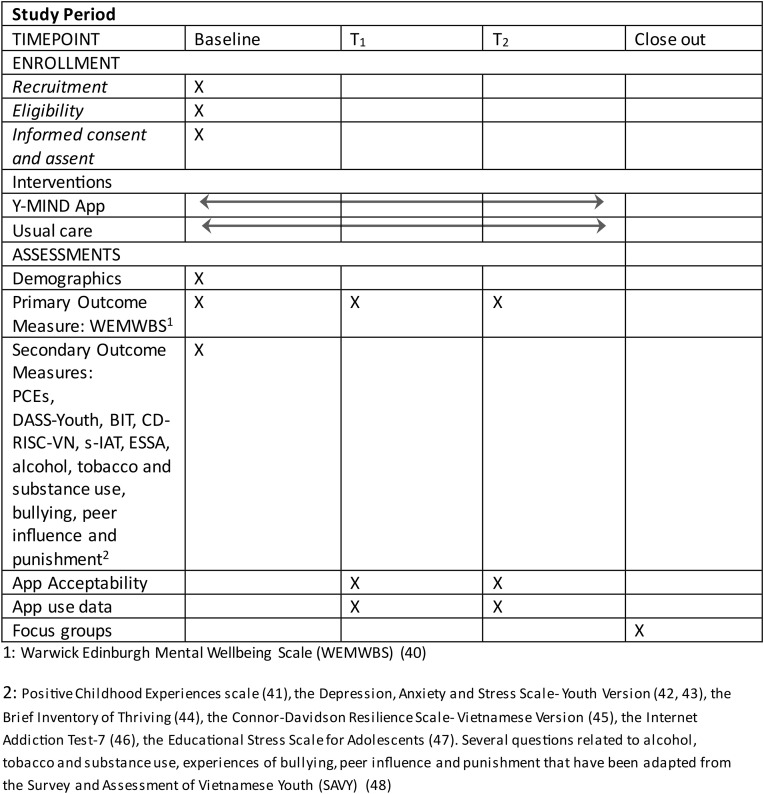
SPIRIT schedule of enrollment, intervention and assessment.

Phase 2 of the study consists of a mixed methods, hybrid type 2 study that will: 1) test and evaluate an implementation strategy to promote uptake and acceptability of the app-based intervention (Y-MIND) among youth in secondary schools and to encourage scale-up across Vietnam; and 2) assess the effectiveness of the intervention on outcomes related to youth well-being, resilience, mental health and related risk factors. Phase 2 will take place in three provinces in Northern Vietnam (Hanoi, Thai Nguyen and Hung Yen). The study process is depicted in the SPIRIT Schedule of Enrollment, Intervention and Assessment (Fig 1). Ethics approval for this study was obtained by the Behavioural Research Ethics Board (BREB) at the University of British Columbia, Canada [H24-01700] and the Institutional Review Board (IRB) of the Institute of Population, Health and Development in Vietnam [2024/PHAD/YPRIME-01-01].Written consent will be obtained from the parents of participants, and written assent will be obtained by youth participants in the study as they are under the age of 18 years.

### Intervention

The intervention, which will be introduced to students in Grade 10, combines two complementary evidence-based approaches: 1) Life Skills Education (LSE) and 2) self-management skills for depression. The first approach draws on the domains identified in the WHO Skills for Health [32] framework and uses the WHO’s *LSE for Children and Adolescents in Schools: Introduction and Guidelines to Facilitate the Development and Implementation of Life Skills Programmes* as a reference point [33]. The second draws on the *‘Dealing with Depression: Antidepressant Skills for Teens’* [34] self-management program which was developed in Canada and provides guidance on self-management to promote coping skills for youth [35]. Although the program was designed for youth experiencing depression, it includes skills that are relevant at a population level to promote well-being and prevent mental ill health. Both programs employ the principles of Cognitive Behavioural Therapy by teaching practical and emotional skills and promoting self-efficacy (CBT).

During Phase 1 (to be described in detail elsewhere), we worked with V-YAC members to co-design an app-based intervention (Y-MIND) that includes six modules based on the LSE and self-management skills domains (Table 1). We originally proposed five modules and added a sixth (Social Media and Well-being) based on input from the V-YAC. Each module includes several lessons and interactive activities (e.g., quizzes, reflection exercises) to allow users to apply the skills they are learning. The lessons also include vignettes, presented as comics, featuring characters who are experiencing related challenges and implementing the skills described in the app to address them. Y-MIND includes several additional features, including a daily mood check-in, guided box breathing exercise, planner and journal. The app also uses gamification to boost engagement via missions and challenges where users can earn points to buy accessories for their avatar. Youth users are free to explore the app at their own pace. All app features, including components of the lessons, were informed by the V-YAC to promote appropriateness and acceptability among secondary school youth in Vietnam.

**Table 1:** Y-MIND app modules and components.

**Problem solving and decision-making**	**Coping with stress and emotions**
• Steps for solving problems and making difficult decisions• Navigating peer influence	• Understanding emotions • Coping with difficult emotions • Coping with stress: time management • Coping with stress: mindfulness
**Effective communication**	**Goal Setting**
• Assertive communication • Tips for talking to adults • Empathy and empathetic communication • Active listening	• Steps for settings goals • Tips for sticking with goals • Using failure as an opportunity to try again
**Realistic Thinking**	**Social media and well-being**
• Negative, positive and realistic thinking • Identifying and challenging negative thoughts and “thinking traps”	• Critical thinking and social media • Dealing with social comparison and avoiding Fear of Missing Out (FOMO) • Creating healthier social media habits

### Study setting

The study will be undertaken among Grade 10 students at four secondary schools in three provinces in the Northern region of Vietnam (Hanoi, Thai Nguyen, and Hung Yen), for a total of twelve study sites. Participating schools will be chosen purposively in collaboration with the Hanoi Department of Education and Training. Participating secondary schools will be selected to include two urban and two rural settings within each province, based on the willingness of school officials to participate in the study and in schools with sufficient numbers of Grade 10 students to ensure an adequate sample size (see below).

### Participants

**Fig 2:**
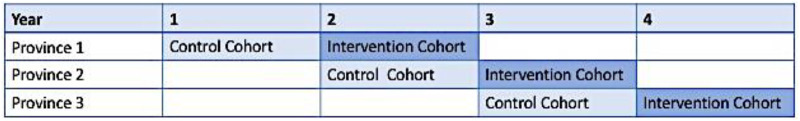
Phase 2 timeline.

Participants will include Grade 10 (15-year-old) students attending participating secondary schools. To reduce the risk of bias as students age, each province will enroll two cohorts in successive years with each cohort followed for one year; the first cohort will not receive the intervention and will yield pre-intervention (control) data, while the second cohort will receive the intervention and yield post-intervention data (Fig 2). Our target is to recruit n = 150 students per school, with n = 75 students in each of the intervention and control cohorts. This number is based on an estimated average of 3600 students across the twelve participating schools, and a target recruitment rate of 25%. Details of sample size and power calculations are provided below. Recruitment rates in Vietnamese studies tend to be higher compared with other jurisdictions. In previous studies among adult populations in community settings, we exceeded our target recruitment rates substantially [36,37]. Given the support of schools and school staff in this study, high rates of smartphone use [38] and engagement with apps among youth in Vietnam, along with the involvement of Vietnamese youth in the co-design of the intervention and app, we estimate that 25% is a feasible target rate for recruitment.

### Recruitment and assent/consent process

We will meet with the school health staff, head teachers, and school principals to inform them about the study and the Y-MIND intervention, and to engage two staff members from each school as study liaisons to help with recruitment.

To recruit students, study team members from the Institute of Population, Health and Development (PHAD) in Vietnam, will make a presentation to Grade 10 students at all participating schools. Following the presentation, the study team will provide a QR code to an online form where interested students can provide their name and their preferred method of communication (email and/or mobile phone number). To enroll in the study, each student’s parent or guardian must provide informed consent, while the student participant must provide informed assent, which recognizes the importance of involving youth in decision-making about their research involvement despite not being able to legally give informed consent [39]. Teachers will assist with following up with parents about their child’s interest in the study and about the processes of informed consent and assent and the study team will distribute paper copies of the informed consent form for parents and the assent form for students. Students and parents will also be provided with an email address and phone number for the PHAD study team to allow them to ask any questions about the study. Upon completion of assent and consent forms, a link to the baseline questionnaire will be sent to the students using their preferred method of communication.

### Inclusion and exclusion criteria

Inclusion criteria are youth enrolled in grade 10 of participating secondary schools, who have access to a smartphone, who provide assent to participate and whose parents provide consent for their child to participate.

Participants will be ineligible to participate if they are not in grade 10, not enrolled in participating secondary schools, they do not provide assent or if their parents do not provide informed consent for their child to participate.

### Study procedure

Control and intervention cohorts will be enrolled in each school in successive years. The control cohort will be recruited first, and will be given the outcome assessments survey at baseline, 6 months, and 12 months, but will not receive access to the app. The outcome assessment survey will be administered using Qualtrics survey software [40]. For ethical purposes, we will provide the control cohort access to the app after they have completed their final outcome assessment survey, but they will no longer provide outcome data, and any in-app usage data will be excluded from the final analysis.

The intervention cohort, recruited the year after the control cohort, will have full access to the app and will complete the same outcome measures at baseline, 6 and 12 months. Their app-usage data, as described below, will be included in the final analysis. Recruitment of the first control cohort began in January 2025, with intervention cohort recruitment taking place in January 2026. Recruitment will be completed in December 2027, with data collection, including post- intervention focus groups discussion as described below, completed in February 2028. Final results are expected to be disseminated in the fall of 2028.

### Outcomes

#### Implementation outcomes.

Quantitative implementation indicators assessed will include uptake (number of participants enrolled in the study) and retention (number of enrolled participants who continue using the app across the 12-month period). To assess app acceptability, we will ask participants to respond to five questions using a 5-point Likert scale ranging from “strongly disagree” to “strongly agree”. These questions were adapted from existing app acceptability measures [41] and simplified based on input from the V-YAC: *1) I like using this app; 2) This app is easy to use; 3) I like the way this app looks; 4) I would be likely to recommend this app to a friend; 5) This app will help me to learn and practice strategies to promote my mental well-being in the next week.*

We will also measure engagement with the app via in-app data. This includes the frequency of app log-in and duration of app engagement (how long are users using the app each time they open it) and the frequency and duration of feature engagement (how long are users interacting with each feature/lesson/activity). In-app data will be collected at two time-points during the intervention delivery to account for the relatively long intervention course and in recognition that natural events in students’ lives (exam periods, holidays, etc.) may lead to variations in app engagement (Fig 1).

#### Clinical outcomes.

We will collect demographic data at baseline only. The primary outcome will be mental well-being, measured using the Warwick Edinburgh Mental Wellbeing Scale (WEMWBS) [42], a 14-item scale that uses positively worded statements to assess feeling and functioning aspects of mental well-being in the general population.

Secondary outcome measures include: the Positive Childhood Experiences scale [43,44], the Depression, Anxiety and Stress Scale- Youth Version [45,46], the Brief Inventory of Thriving [47], the Connor-Davidson Resilience Scale- Vietnamese Version [48], the Internet Addiction Test-7 [49], the Educational Stress Scale for Adolescents [50]. Several questions related to alcohol, tobacco and substance use, experiences of bullying, peer influence and punishment that have been adapted from the Survey and Assessment of Vietnamese Youth (SAVY) [51].

All measures and the complete suite of outcome measures were reviewed by V-YAC members for face validity, clarity and appropriateness. We made minor adjustments to the survey based on their feedback. We then pilot tested the full survey with fifteen Grade 10 students who responded to questions about the survey’s clarity and feasibility. Based on the advice of the V-YAC and pilot testers and due to the length of the questionnaire, we have added cartoons with encouraging messages throughout the survey to promote completion of the survey.

Outcome assessments will be conducted using a Qualtrics online survey, which enrolled students may complete on their own devices or at school at each time point. This will ensure that outcome data collection is not dependent on app use. The 6- and 12-month data collection will help us to understand factors influencing engagement over time and immediate and long-term effects of the intervention. We will collect quantitative data at baseline, 6 months and 12 months for each cohort.

We will also include the Patient Health Questionnaire-8 (PHQ-8) [52] and General Anxiety Disorder-7 (GAD-7) [53] scales in the app for mental health self-assessment and to allow participants to track their outcomes, but these scores will not be collected as study data as participants will be able to access these measures at any time point for their own self-monitoring. To ensure confidentiality and privacy these scores will not be shared with anyone aside from the participant when they complete the measure within the password protected app. To promote safety participants scoring 10 or above (suggesting moderate to severe depression or anxiety) on either measure will receive an in-app pop-up notification recommending that they seek additional support by talking to a trusted adult and visiting a health care provider. This message will be repeated each time the participant completes the measures and scores a 10 or above. As mental health services in Vietnam are limited and there is evidence of low levels of awareness about where to access mental health care, we will include a list of resources and a guide to seeking mental health support in the app and advice about how to talk to a trusted adult about their mental health.

### Randomization and blinding

Participating schools will be selected purposively as described above and randomization will not occur as this is a quasi-experimental study.

Due to the nature of the intervention blinding will not be possible.

### Sample size and power calculations

Our power analysis is based on outcome comparison at the last visit (12 months). Our target is to recruit n = 150 students per school, with n = 75 students in each of the intervention and control cohorts. Thus, each province will enroll 75*4 = 300 students per cohort with a total of 300*3 = 900 per cohort in the study sample. This number is based on an estimated average of 3600 students across the twelve participating schools, and a target recruitment rate of 25%. The standard deviation of scores across students is assumed to be 6.8 (as observed cross-sectionally in youth aged 13−16 in the UK [42]. The comparison of the WEMWBS scores in the post- versus the pre-intervention groups will have 80% power (assuming a two-sided Z-test at alpha = 0.05) if the true effect of the intervention (i.e., the “detectable difference”) is approximately the 0.9 points averaged across *all* participants, assuming a homogeneous treatment effect across schools. As discussed above, the prevalence of stress and depression has been found to be ~ 40% in this student population. If we assume that the intervention results in a benefit in 30% of students (and no impact in the other 70%), this would require that the benefit in the 30% group be 0.9/ (1–0.7) = 3.0 points on average, which matches to the magnitude of the effect seen in a previous trial of a web-based CBT program to promote mental well-being offered in a general population [54]. The actual power likely will be increased as a result of the analysis accounting for baseline WEMWBS scores, which are expected to be predictive of post-intervention scores. Power may be reduced if the treatment effect is heterogeneous across schools. However, our expectation is that the study will have sufficient power to detect effects pooling over all schools across provinces.

### Statistical analysis

In the initial analysis, the distributional characteristics of the variables will be explored using descriptive statistics. For continuous variables, this will include measures of central tendency (such as the mean, median, and quartiles) and measures of variability (such as the range, standard deviation, and variance). For categorical variables, frequency counts of each category will be reported. These descriptive measures will also support the data cleaning and screening process by helping to identify potential errors related to data entry, handling, or measurement.

The overall analytical strategy for the various types of outcomes in this study will employ Generalized Linear Mixed-Effect Models (GLMMs), which are well-suited for longitudinal and clustered data, with the **school** specified as the clustering variable. These models, previously used in an earlier study [36], are flexible and capable of handling diverse outcome types—including normal, binary, ordinal, and Poisson distributions—while also accounting for correlations among within-subject and/or within-cluster observations.

The primary endpoint of this study—mental well-being as measured by the WEMWBS, a continuous outcome—will be analyzed using a three-level linear mixed-effects regression model, a specific form of GLMMs. The main independent variables in the model will include:

- Visit indicators for 6 and 12 months to account for secular trends,
- Group indicators for the intervention vs. control group,
- Group by visit interaction,
- and baseline WEMWBS scores as fixed effects.

In addition, the models will include random effects for both schools and participants nested within schools to capture variability at both the cluster and individual levels. A random group X school effect will be included to allow for heterogeneity of intervention effects across schools. The estimated coefficients for the group X visit interaction will represent the primary intervention effects of interest.

The same linear mixed effects model will be applied to analyze continuous secondary outcomes. For binary and count secondary outcomes (e.g., the proportion of substance use or the frequency of substance use), we will use mixed-effects logit for longitudinal binary outcomes or mixed-effects log-linear models for longitudinal count outcomes. By correctly specifying the GLMMs, the analysis will allow for testing a broad set of scientific hypotheses, including those central to evaluating the intervention effects. The robustness of the statistical inferences will be ensured through comprehensive model checking and validation procedures.

All analyses will be conducted using SAS 9.4, specifically with the PROC MIXED, PROC GLIMMIX, and/or PROC NLMIXED procedures.

Subgroup analyses will also be carried out using GLMMs on stratified subsets of the data. To ensure we understand differences in implementation and clinical outcomes across diverse sub-populations of youth, we will conduct subgroup analyses according to demographic features including gender and gender identity, sexual orientation, rural vs. urban location, age, socio-economic status, ethnicity, and provinces.

Missing data is a common issue in most studies and is an important practical consideration when implementing the statistical models described above. A key advantage of using GLMMs is their ability to utilize all available data, including observations with missing outcome values due to attrition. These models yield valid inferences under the assumption that data are Missing at Random (MAR)—a less restrictive assumption than Missing Completely at Random (MCAR). Under the MAR assumption, missingness is considered to depend on observed data rather than unobserved values. If the nonresponse behavior can reasonably be explained by the observed data, the missing values will be treated as MAR. However, if there is concern that the missingness may still be nonrandom even after conditioning on observed information, the potential bias will be evaluated using selection models [55–58].

### Qualitative methods

To assess implementation outcomes, we will conduct focus group discussions (FGDs) with 6–8 youth participants at each school at the 12-month mark for each intervention cohort to explore their experiences with the app, focusing on usability, appropriateness, and acceptability of app use. FGD questions will be co-designed with the V-YAC to ensure they capture appropriate themes that reflect factors influencing user satisfaction, engagement and acceptability from the perspective of youth participants. Through FGDs we will explore in-depth factors influencing acceptability and appropriateness to understand both positive and negative experiences with the intervention content and interface. This will allow us to assess appropriateness and acceptability of the app and identify areas for refinement. We will distribute invitations to students inviting them to participate in a focus group using the contact information provided to the study team to invite their participation in the focus groups. We will aim to include students representing all genders and with different levels of engagement with the app to understand factors influencing engagement levels based on preliminary analysis of in-app data. We will generate lists of student participants according to gender and app use level (high, average and low) and will randomly select students from each group to receive invitations. We will repeat this process until we have recruited 6–8 from each school.

We will also conduct semi-structured interviews with school staff (e.g., principals, school health staff, teachers) at each school (N = 36 across all schools) to understand factors affecting implementation and potential scale-up of this intervention. Interview questions will explore the attitudes of school staff related to the intervention (e.g., perceived impact on youth and school environment), perceived factors influencing implementation and scale-up (e.g., what would facilitate sustained implementation in their school, what resources would be needed for scale-up to other secondary schools). We will use thematic analysis [59] to analyse FGD and semi-structured intervention data.

### Data management

All outcome measures will be administered through Qualtrics [40], a secure web-based survey database management application. The dataset will then be divided into two parts: one containing only identifiable information (such as names and school) and another containing the remaining data for analysis. The dataset used for analysis will be stored on the University of British Columbia’s (UBC) secure Microsoft OneDrive server. The portion containing identifiable information will be stored on a secured laptop at PHAD’s office. Paper copies of transcripts and notes taken during data qualitative data collection will be stored in a locked cabinet in a secured office. Electronic copies of transcripts and audio recordings collected during the qualitative data collection will be stored on a secured laptop. All app data will be stored on the PHAD secure server. Access to these databases will be limited only to authorized team members (Co-PIs, statisticians, UBC and PHAD research coordinators). All data (excluding app data) will be stored on Canada’s Federated Research Data Repository (FRDR) [60] for future open access initiatives. Local copies of all data will be stored on the various servers and then destroyed after five years, according to Vietnamese data retention policy.

### Confidentiality

After the full dataset is complete, each record will be assigned a unique code. The dataset will then be divided into two parts: one containing only identifiable information (such as names and school) and another containing the remaining data for analysis. Both parts will include the unique code for each record, allowing us to cross-reference them if necessary. The dataset without identifiable information will be used for analysis, while the file containing identifiable data will be stored in a secure, password-protected and encrypted folder on PHAD’s server. Access to this file will be strictly limited to members of the study team.

All potentially identifying information (e.g., names or initials, location of residence, specific occupation or school, etc.) will be excluded from study datasets at the time of transcription. No identifying features will be attributed to study participants in any resulting publications, presentations or other knowledge dissemination activities. In transcripts and when making use of illustrative quotations in the reporting of study findings, participants will be identified with a randomly assigned number (e.g., P1, P2) which will not be associated with the participants’ identity.

To register for app use, we will collect participants’ name, email, phone number, and school name. The raw data collected through the app will include this information, however, the identifying information will be removed during analysis. The app data will be stored on PHAD’s secure server and backed up weekly. PHAD will assign one researcher the responsibility of removing identifying information from the study datasets. The Y-MIND app will use end-to-end 256-bit encryption to ensure privacy and users’ data will never be shared with third parties without explicit consent.

Once the app is available on the Apple App Store and Google Play Store, it can be downloaded and used by anyone. However, our focus is solely on data from registered study participants. Using the information provided during registration, such as name, email, phone number, and school name, we are able to identify eligible participants. Only the app data from these verified participants will be retained and used for further study activities.

### Retention strategies

To promote retention during the 12-month study period, each participant will receive a small gift at the end of the 12-month study period, valued at approximately 50,000 VND (CAD $ 2.5). The gift may be a tote bag, a notebook or a pencil case with the study logo. This process was determined based on the advice of school leadership.

## Discussion

The Y-PRIME study responds to a critical gap in mental health and well-being promotion and risk factor prevention for adolescents in Vietnam. Youth in Vietnam experience high levels of psychosocial stress and encounter numerous challenges and exposures that might act as risk factors for poor mental health in the immediate and long-term. The potential of using app-based mental health supports has been identified as an acceptable approach for youth in Vietnam [9], but the previous lack of engagement with end users has created a barrier to successful uptake of these approaches [29]. Similarly, low levels of mental health awareness in Vietnam, including among school teachers and health staff, suggest that an intervention delivered via a mobile app could provide much-needed support to youth in school without requiring substantial additional resources or training [7]. We therefore hypothesize that a population-level, youth co-designed skills-based intervention, delivered via an app, will show positive implementation and clinical effectiveness outcomes.

This study builds on over a decade of partnership between Vietnamese, Canadian and Australian researchers and with policy makers in Vietnam to enhance the delivery of mental health care in the country [61]. This current study includes the engagement of key stakeholders including policy makers at the Departments of Education and Training, school staff and youth with lived experience. This partnership has the potential to promote successful implementation and to lay the groundwork for scale-up throughout the country. In the context of our broader program of research, the Government of Vietnam has demonstrated an increased interest and commitment to the use of digital technologies to deliver mental health care. This commitment was further strengthened during the COVID-19 pandemic as, like much of the world, the necessity of using digital technologies has illuminated its long term potential in terms of expanding availability and accessibility of care [62]. The integrated knowledge translation [61] approach taken throughout this study will promote long term engagement at the school and policy level, with the goal of achieving buy-in to support the scale-up of the intervention following the study. The co-design process with Vietnamese youth will help to promote the appropriateness and acceptability of the Y-MIND app among youth in Vietnamese schools, as it is directly tailored to respond to their concerns and experiences.

This study has several potential limitations. The first is the risk of attrition given the 12-month period of enrollment. We expect that the gap in mental health promotion and well-being support for youth, an increasing awareness of and interest in mental health and well-being among youth, and the fact that the app was co-designed with Vietnamese youth will help to promote sustained engagement in the study. Participants in the control cohorts will receive access to the app following their 12-month participation in the study.

There is increasing interest in the Asian region in school-based mental health programs. At the time of writing, no such programs exist in Vietnamese secondary schools. However, there is a small chance that such programs might be introduced during the study period. Should this occur, we will communicate with our partners at the Department of Education and Training and within the participating schools to ensure that we understand the parameters of any newly introduced programs. Implementation research, by nature, must be responsive and adaptive to the real-world settings in which it takes place, and we will ensure we are aware of and responsive to any conditions that might change the study context and influence the results of the study.

An additional potential limitation is that this study takes place in only three provinces of Vietnam, with all study provinces located in the north of the country. The Government of Vietnam is currently undertaking a restructuring process that includes a merger of provinces, reducing them by approximately half. The study provinces were selected for feasibility due to the relationship with the Hanoi Department of Education and Training and due to resource limitations. This means that study results may not be generalizable to students or schools in other regions of Vietnam, which is culturally and linguistically diverse. Despite the narrow geographic scope of the study, however, the app-based intervention was co-designed with input from youth living across Vietnam, capturing the perspectives of youth from southern, central and northern regions of the country. Our hope is that should the current study prove effective we will be able to undertake further testing and expand our collaboration with policy partners in different regions to inform the scale-up of the app across the country.

Finally, although we included Vietnamese validated versions whenever possible, many of the outcome measures were developed in Western contexts. To address this potential limitation, we worked with the V-YAC to assess the face validity, clarity and appropriateness or the outcome measures. Based on their feedback, we adjusted the wording and changed some of the measures used to ensure they were meaningful and easily understood by Vietnamese youth. We then pilot tested the outcome measures survey among 15 Grade 10 youth in Hanoi to assess the survey’s clarity and feasibility. Based on the comments from the V-YAC and pilot testing about the length of the survey and on the advice of these youth, we included cartoons with encouraging messages throughout the survey to sustain engagement with the survey.

## Conclusions

The Y-PRIME study takes place in the context of increased global recognition of the urgency of supporting youth mental health, well-being and resilience. It also aligns with the rapid expansion of digital technologies to deliver mental health promotion, mental ill health prevention and care interventions. The need for evidence-based approaches that are informed by youth perspectives, particularly from LMICs, is pressing. Similarly, the importance of implementation science for promoting real-world uptake of evidence-based approaches is clear. Understanding the potential for clinical effectiveness and successful implementation of the Y-MIND app will contribute important evidence to the fields of youth mental health, digital health and implementation science. Should the results of the study be positive, they can inform scale-up and expansion of the Y-MIND intervention in Vietnam and its potential adaptation for other settings.

We would like to acknowledge the essential contributions of Phuong Kieu, Van Linh Le, Hoang Tuan Nguyen, Linh Tu Nguyen, Phu Nguyen, Van Pham, Cat Pham, Ngoc Linh Tran, Thao Linh Tran, Lien Tran, Viet Anh Hoang and Kieu Anh Vu – all members of the Vietnamese Youth Advisory Council – in co-designing the Y-MIND app and intervention.

## Supporting information

S1 File SPIRIT checklist. (DOCX)

S2 File Ethics protocol. (DOCX)
