# Supplementary material for: Promoting mental well-being among secondary school students in Vietnam using the Y-MIND app: A protocol for a hybrid type 2, sequence pre-post, quasi-experimental study
Source: PLoS One. 2025 Oct 7;20(10):e0332875. doi: 10.1371/journal.pone.0332875 (PMC12503240; doi:10.1371/journal.pone.0332875)
Supplement: S2 File — (DOCX) [file pone.0332875.s002.docx]

| \| Date: 11/4/2024, 10:07:28 AM \|  \|  \| \| --- \| --- \| --- \| |
| --- | --- | --- | --- |
| \| \|  \|  \| *The University of British Columbia Office of Research Ethics* ***Behavioural Research Ethics Board*** *Suite 102, 6190 Agronomy Road Vancouver, BC V6T 1Z3* \| \| --- \| --- \| --- \| \|  \| \| \| \| \| --- \| --- \| --- \| --- \| --- \| --- \| --- \| \| ***H24-01700*   Y-PRIME P2   (Version 1.0)** \| \| \| **Principal Investigator: Raymond W. Lam** \| \| \| **1. Principal Investigator & Study Team - Human Ethics**[[View Form]](https://rise.ubc.ca/rise/app/portal/smartform/edit?Project=com.webridge.entity.Entity%5BOID%5B100ACDEE22CB11EFFC91E27501565000%5D%5D&doValidation=False&Mode=smartform&WizardPageOID=com.webridge.entity.Entity%5BOID%5B6A65CFE3D889274EBA8A159920B51120%5D%5D) \| \| \| ***1.1. Principal Investigator*** \| \| **Last Name** \| **First Name** \| **Employer.Name** \| **Email** \| \| --- \| --- \| --- \| --- \| \| Lam \| Raymond W. \| Psychiatry \| r.lam@ubc.ca \| \| \| *Enter Principal Investigator's secondary appointments or affiliations (including Health Authorities), if applicable:* \|  \| \| ***1.2. Primary Contact*** \| \| **Last Name** \| **First Name** \| **Rank** \| \| --- \| --- \| --- \| \| Tran \| Rosie \| Research Assistant \| \| \| ***1.3A. Co-Investigators - Online Access*** \| \| **Last Name** \| **First Name** \| **Institution/Department** \| **Rank** \| **TCPS2** \| \| --- \| --- \| --- \| --- \| --- \| \| O'Neil \| John \| Simon Fraser University \| Professor \| yes \| \| Hayashi \| Kanna \| UBC/Medicine, Faculty of/Medicine, Department of/Division of Social Medicine \| Affiliate Associate Professor \| yes \| \| Michalak \| Erin \| UBC/Medicine, Faculty of/Psychiatry \| M&P Staff \| yes \| \| Samji \| Hasina \| Simon Fraser University \| Assistant Professor \| yes \| \| Barbic \| Skye \| UBC/Medicine, Faculty of/Occupational Science and Occupational Therapy \| Associate Professor \| yes \| \| Murphy \| Jill \| Simon Fraser University \| Non-UBC Faculty Member \|  \| \| \| ***1.3B. Describe each Co-I's role in study, e.g. statistician, supervisor, adviser, student etc. Ensure individual is entered in Box 1.3A*** \| Dr. Michalak: Expertise in patient engagement and participatory app development Dr. Hayashi: Expertise in community-based research in Asia Dr. Barbic: Expertise in youth mental health and youth engagement Dr. O'Neil: Expertise in mental health research in Vietnam Dr. Murphy: Expertise in mental health research in Vietnam and digital mental health Dr. Samji: Expertise is youth mental health, measurement \| \| ***1.4A. Additional Study Team Members - Online Access*** \| \| **Last Name** \| **First Name** \| **Institution/Department** \| **Rank** \| **TCPS2** \| \| --- \| --- \| --- \| --- \| --- \| \| Evans \| Vanessa \| UBC/Medicine, Faculty of/Psychiatry \| Research Assistant \| yes \| \| Tran \| Rosie \| UBC/Medicine, Faculty of/Psychiatry \| Research Assistant \| yes \| \| \| ***1.4B. Describe each Additional Study Team Members' role in study, e.g. staff, research assistant etc.*** \| Vanessa Evans is a Research Manager with our team and will provide administrative and research support throughout the study.  Rosie Tran is the primary Project Coordinator for Y-PRIME, providing full-time/day-to-day administrative and research support for the study. \| \| ***1.5A. Additional Study Team Members - No Online Access*** \| \| **Last Name** \| **First Name** \| **Institution / Department** \| **Rank / Job Title** \| **Email Address** \|  \| \| --- \| --- \| --- \| --- \| --- \| --- \| \| Chau \| Leena \| Simon Fraser University, Faculty of Health Sciences \| PhD Candidate \| leena_chau@sfu.ca \|  \| \|  \|  \|  \|  \|  \|  \| \| Vu Cong \| Nguyen \| nstitute of Population, Health and Development, Hanoi \| Deputy Executive Director \| nguyen@phad.org \|  \| \|  \|  \|  \|  \|  \|  \| \| Minas \| Harry \| University of Melbourne, Global and Cultural Health Unit \| Associate Professor \| h.minas@unimelb.edu.au \|  \| \|  \|  \|  \|  \|  \|  \| \| \| ***1.5B. Describe each Additional Study Team Members' (no online access) role in study, e.g. external supervisor, consultant etc.*** \| Dr. Nguyen is the co-Principal Investigator and will lead the study implementation in Vietnam  Dr. Minas has extensive experience and expertise in mental health policy development and research in Vietnam. He is a co-investigator on this study.  Ms. Chau is a PhD candidate at SFU who is studying digital mental health in the country. She is a co-investigator on this study. \| \| ***1.6. Tri Council Policy Statement (TCPS) Tutorial*** *Have all research personnel completed the required TCPS2 tutorial:* \| Yes \| \| ***1.7. Project Title*** *Enter the title of this research study as it will appear on the certificate. Title given****must match****the title on all study documents.* \| Youth Promotion of Resilience Involving Mental E-health (Y-PRIME) - Phase 2 \| \| ***1.8. Project Nickname*** *Enter a nickname for this study. What would you like this study to be known as to the Principal Investigator and study team?* \| Y-PRIME P2 \| \| **2. Study Dates and Funding - Human Ethics**[[View Form]](https://rise.ubc.ca/rise/app/portal/smartform/edit?Project=com.webridge.entity.Entity%5BOID%5B100ACDEE22CB11EFFC91E27501565000%5D%5D&doValidation=False&Mode=smartform&WizardPageOID=com.webridge.entity.Entity%5BOID%5B38B1507DDE40DC4B84FC8F89C18C27DF%5D%5D) \| \| \| *You plan to start collecting data immediately after obtaining ethics and any other required approvals* \| no \| \| *You plan to start data collection at a later date i.e., 2 months or more after approvals are obtained. Click the calendar icon below to select the dates.  Estimated start date:* \| 9/1/2024 \| \| ***2.1.B.*** *Estimated end date:* \| 1/31/2028 \| \| ***2.2.A. Types of Funds*** *Please select the applicable box(es) below to indicate the type(s) of funding you are receiving to conduct this research.****You must then complete section 2.3 and/or section 2.4 for the name of the source of the funds to be listed on the certificate of approval.*** \| Grant \| \| ***2.2.B.****For Industry Sponsored studies, please provide a sponsor contact.* \|  \| \| ***2.3.A.****Research Funding Application/Award Associated with the Study that was Submitted to the UBC Office of Research Ethics* \| \| **UBC Number** \| **Title** \| **Sponsor** \|  \| \| --- \| --- \| --- \| --- \| \| F22-01650 \| Youth promotion of resilience involving mental e-health (Y-PRIME) \| Canadian Institutes of Health Research (CIHR) \|  \| \|  \|  \|  \|  \| \| \| ***2.3.B.****Which institution is administering the funds, if not UBC or UBC affiliated institution?* \|  \| \| ***2.4.A.****Research Funding Application/Award Associated with the Study not listed in question 2.3.* \| \| **UBC Number** \| **Title** \| **Sponsor** \|  \| \| --- \| --- \| --- \| --- \| \| \| ***2.4.B.****Please enter any applicable information about your funding which is not already shown in Box 2.3A or 2.4A (including funding applied for but not yet received).* \|  \| \| ***2.5.A.****Is this a DHHS grant?* \| no \| \| ***2.5.B.****If yes, please select the appropriate DHHS funding agency from the selection box.* \| \| **DHHS Sponsor List:** \| **Order:** \| **Active:** \| \| --- \| --- \| --- \| \| \| ***2.6. Study Related Conflict of Interest*** *Conflicts of Interest (COIs) in research are situations where someone's personal interests (financial, career, or other) could compromise or could be perceived to compromise the objective conduct of research or integrity of the data.  Conflicts of interest  can arise naturally from an Investigator's engagement inside and outside the University, and the mere existence of a COI or the perception of a COI does not necessarily imply wrongdoing on anyone's part. Nonetheless, real and perceived COI must be recognized, disclosed, and assessed. This question asks Investigators to disclose COIs that may relate to the research study that is the subject of the REB application.  Do the Principal Investigator, Co-Investigators and/or their related parties have any personal interest(s) that could compromise or reasonably be perceived to compromise the objective conduct of the research or the integrity of the data generated by the study? Personal interests may include business, commercial or financial interests, dual roles (e.g. PI and Doctor), as well as personal matters and career interests.* \| no \| \| **4.A. Study Type - (Boxes 4.1 to 4.2C)**[[View Form]](https://rise.ubc.ca/rise/app/portal/smartform/edit?Project=com.webridge.entity.Entity%5BOID%5B100ACDEE22CB11EFFC91E27501565000%5D%5D&doValidation=False&Mode=smartform&WizardPageOID=com.webridge.entity.Entity%5BOID%5BAFDE35BE2D9AEF49A3FECBC661048CCD%5D%5D) \| \| \| ***4.1. Application Type*** *Indicate whether your application is Clinical or Behavioural.* \| Behavioural \| \| ***4.2.A.****Institutions and Sites for Study (including study team members' institutional affiliations under which this research is being conducted)* \| \| **Institution** \| **Site** \| \| --- \| --- \| \| UBC \| Vancouver (excludes UBC Hospital) \| \|  \|  \| \| BC Centre for Disease Control \| BC Centre for Disease Control \| \|  \|  \| \| Vancouver Coastal Health (VCHRI/VCHA) \| Centre for Brain Health \| \|  \|  \| \| Providence Health Care \| St. Paul's Hospital \| \|  \|  \| \| \| ***4.2.B.****Non-UBC Institutions and Sites for Study (including study team members' institutional affiliations under which this research is being conducted)* \| \| **Institution** \| **Site** \| \| --- \| --- \| \| Simon Fraser University \| Vancouver \| \|  \|  \| \| \| ***4.2.C.****Please enter any other locations where the research will be conducted under this Research Ethics Approval (e.g., Name of privately owned clinic, community centre, school, classroom, participant's home, in the field - provide details).* \| Institute of Population, Health and Development, Hanoi, Vietnam will lead the study implementation in Vietnam. We are currently working with Hanoi’s Department of Education and Training to identify participating schools and will update when we receive the names of participating schools \| \| **4.B. Behavioural Study Type - (Boxes 4.2D to 4.6)**[[View Form]](https://rise.ubc.ca/rise/app/portal/smartform/edit?Project=com.webridge.entity.Entity%5BOID%5B100ACDEE22CB11EFFC91E27501565000%5D%5D&doValidation=False&Mode=smartform&WizardPageOID=com.webridge.entity.Entity%5BOID%5B4545E00E9CDB6344A0BAC3F0C07DC900%5D%5D) \| \| \| ***4.2.D. Roles of Study Sites and Institutions*** \| \| **Study Site:** \| **Accessing Records or Charts:** \| **Analysing Data or Utilizing Lab Space:** \| **Recruiting Participants:** \| **Team Member Affiliations:** \|  \| \| --- \| --- \| --- \| --- \| --- \| --- \| \| UBC - Vancouver (excludes UBC Hospital) \| no \| yes \| no \| yes \|  \| \|  \|  \|  \|  \|  \|  \| \| BC Centre for Disease Control - BC Centre for Disease Control \| no \| no \| no \| yes \|  \| \|  \|  \|  \|  \|  \|  \| \| Simon Fraser University - Vancouver \| no \| no \| no \| yes \|  \| \|  \|  \|  \|  \|  \|  \| \| Vancouver Coastal Health (VCHRI/VCHA) - Centre for Brain Health \| no \| yes \| no \| yes \|  \| \|  \|  \|  \|  \|  \|  \| \| Providence Health Care - St. Paul's Hospital \| no \| no \| no \| yes \|  \| \|  \|  \|  \|  \|  \|  \| \| \| ***4.3.A.****If this proposal is closely linked to any other proposal previously/simultaneously submitted, enter the Institution or Health Authority name and associated Research Ethics Board study number of that proposal.  Institution Name:* \| UBC \| \| *REB study number:* \| H23-01104 \| \| ***4.3.B.****If applicable, please describe the relationship between this proposal and the previously/simultaneously submitted proposal listed above.* \| The previous study (REB H23-01104) is phase 1 of our study and focuses on engagement with youth in Vietnam to build capacity for leadership in mental health and to co-design an app-based intervention to promote mental health and well-being among youth in Vietnam. In Phase 2, we will test the mobile app developed in Phase 1 among Vietnamese youth in secondary school. \| \| ***4.3.C.****Have you received any information or are you aware of any rejection of this study by any Research Ethics Board? If yes, please provide known details and attach any available relevant documentation in Box 9.7.* \| no \| \| *Please provide known details:* \|  \| \| ***4.4.A.****External peer review details:* \| External peer review took place under the Canadian Institutes for Health Research (CIHR) team grants program, as part of a competitive funding call led by the Global Alliance for Chronic Diseases (GACD) entitled “A life course approach to reducing and preventing common non-communicable disease risk factors”. \| \| ***4.4.B.****Internal (Institution or hospital) peer review details:* \| N/A \| \| ***4.4.C.****If this research proposal has not received any independent scientific/methodological peer review, explain why no review has taken place.* \| N/A \| \| *Participant Vulnerability* \| Medium \| \| *Research Risk* \| Low \| \| ***4.5.B.****Provide explanations for the assessments of research risk and participant vulnerability reported above.* \| In phase 2 of Y-PRIME, we will be testing an app-based intervention co-designed by Vietnamese youth, the research team, and a Vietnamese software development company with Vietnamese secondary school students. Youth, in general, may be considered a somewhat vulnerable group. The intervention takes a population-level approach to enhancing life skills and self-management skills that may be beneficial to all youth regardless of their mental health status. As this is a universal intervention, all students in grade 10 of participating secondary schools will be eligible to participate regardless of mental health and well-being status, as measured by standardized outcome measures (see below for details). Students who are identified at baseline as having symptoms of moderate or severe depression and/or anxiety will be referred to appropriate care (e.g. primary care centres or emergency services). The app will also have recommendations to help youth participants reach out to local mental health support facilities if they need extra support. We therefore consider this study phase to be low risk and do not anticipate that participation in this study will pose a substantial risk to youth participants. As described below, we will ensure that all data collected via the app are accessible only to the study team and that the app adheres to appropriate protocols related to data privacy and security. \| \| ***4.5.C.****Does your application fall under minimal risk (i.e., was it assigned an overall risk level of 1 or a blue box on the minimal risk matrix above)?* \| yes \| \| **4.C. Behavioural Study Type - (Boxes 4.7 to 4.8)**[[View Form]](https://rise.ubc.ca/rise/app/portal/smartform/edit?Project=com.webridge.entity.Entity%5BOID%5B100ACDEE22CB11EFFC91E27501565000%5D%5D&doValidation=False&Mode=smartform&WizardPageOID=com.webridge.entity.Entity%5BOID%5B39FF2BA001749C4F9EB436A68E7A6551%5D%5D) \| \| \| ***4.7.A Creation of a Research Database or Registry*** *Does this study involve the creation of a research database or registry with a local custodian for future unspecified research?* \| no \| \| ***4.7.B.****Is the purpose of this application exclusively to obtain approval for the creation of a research database or registry? [Note: if the creation of the database or registry is part of a bigger project also included in this application, you must answer "no" below].* \| no \| \| ***Survey Research*** *Is this a****minimal risk****study exclusively using a survey for data collection?* \| no \| \| ***Secondary Use*** *Is this a****minimal risk****study exclusively analyzing previously collected data?* \| no \| \| **5. Summary of Study and Recruitment - Behavioural Study**[[View Form]](https://rise.ubc.ca/rise/app/portal/smartform/edit?Project=com.webridge.entity.Entity%5BOID%5B100ACDEE22CB11EFFC91E27501565000%5D%5D&doValidation=False&Mode=smartform&WizardPageOID=com.webridge.entity.Entity%5BOID%5BCF8809E32AAAF54EA0DCF6A7247A819C%5D%5D) \| \| \| ***5.1.A.****Provide a brief statement about the project written in lay language. Do not exceed 100 words and do not cut and paste directly from the study proposal.* \| The goal of Y-PRIME study is to promote mental well-being and address risk factors for poor mental health among youth in Vietnam at a population level. In Phase 1, the study convened a Vietnam Youth Advisory Council (V-YAC) to adapt and co-design an app-based life skills and self-management intervention to ensure the intervention will be culturally appropriate and relevant to Vietnamese youth. In Phase 2, we will be testing this app with 15-year-olds and will assess outcomes related to implementation, mental health and well-being, risk factor mitigation, and equitable access with a view to scaling up the model across the country. \| \| ***5.1.B.****Summarize the research proposal, including study purpose, hypothesis, study population, and research method.* \| Between 10-20% of the world’s children and youth experience mental health problems; suicide is among the five leading causes of mortality among adolescents. Despite this, youth-oriented mental health promotion, prevention, and support are severely limited, especially in low and middle-income countries (LMICs). Vietnam has a population of 97 million people, 70% of whom are under the age of 35 years. Like many LMICs, Vietnam has extremely limited mental health promotion, prevention and care availability for youth. Life skills-based programs, delivered at a population level via schools, are an evidence-based approach that have the potential to improve mental health, resilience, and well-being among youth in low-resource settings. A 2021 systematic review found that more robust evidence, including implementation research, is needed to support the implementation and scale-up of appropriate interventions in LMICs. Digital health approaches including interventions delivered via mobile apps are also increasingly recognized as an effective, acceptable, and accessible approach to promoting youth mental health and wellness and warrant more research, particularly within LMICs.  The Youth Promotion of Resilience Involving Mental E-health (Y-PRIME) study will respond to the gap in the availability of evidence-based interventions to promote mental health, well-being, and resilience for youth in Vietnam. In Phase 1 of the study, we engaged key stakeholders in the adaptation and co-design of an evidence-based mobile app intervention to be used among secondary school students. This application is for phase 2 of the study in which the mobile app will be tested among secondary school students in Vietnam.  We hypothesize that a youth-informed intervention adapted for the Vietnamese context and delivered via an app will lead to positive implementation outcomes among youth and within the implementation environment. We also posit that it will lead to improved mental health, well-being, resilience, and risk-factor-related outcomes among Vietnamese youth.  Youth, defined by the United Nations as persons aged 15-24 years, are the primary study population in Y-PRIME. Phase 2 will consist of a hybrid type 2 (where both implementation and clinical outcome measures are assessed), quasi-experimental, sequenced pre-post implementation design. In Phase 2, working with partners in the Hanoi Department of Education and Training (DoET), we will engage with school health staff (SHS) and head teachers to recruit 15-year-olds enrolled in grade 10 of participating secondary schools. The study will take place at four schools in each of the three provinces, for a total of twelve study sites. Participating schools will be chosen purposively in collaboration with the Department of Education and Training. To reduce the risk of bias as students age, each province will enroll two cohorts in successive years with each cohort followed for one year; the first cohort will not receive the Y-PRIME intervention and will yield pre-intervention (control) data, while the second cohort will receive the Y-PRIME intervention and yield post-intervention data. We will collect quantitative data at baseline, 6 months and 12 months for each cohort. Our target is to recruit n=150 students per school, with n=75 students in each of the intervention and control cohorts. This number is based on an estimated average of 3600 students across the twelve participating schools, and a target recruitment rate of 25%. Recruitment rates in Vietnamese studies tend to be higher compared with other jurisdictions. In previous studies among adult populations in community settings, we exceeded our target recruitment rates substantially (1,2). Given the support of schools and school staff in this study, the high rates of Smartphone use and engagement with apps among youth in Vietnam, the involvement of Vietnamese youth in the co-design of the intervention and app, we estimate that 25% is a feasible target rate for recruitment. As this is a hybrid implementation and effectiveness study, uptake and engagement of the app among students will be included among our implementation outcomes (measured through back end data collected in the app) and will provide important data about the feasibility of scaling up the app after this study is complete.  Previous studies of youth engagement with app-based life skills interventions show that receiving regular reminders by SMS or in-app notifications promotes engagement (3). Final details of the app’s function, including frequency and mode of delivery of reminders, are currently being developed via the co-design process with our Vietnam Youth Advisory Council, but students will receive frequent reminders to engage with the intervention over the course of twelve months (3). We will also include gamification elements such as having avatars to represent the users in the app. Students can showcase their individuality by changing their astronaut colours and different accessories (hats, pets, costumes, masks). Students will be awarded points and badges in the app for completing each lesson, activity, or challenge (aimed to help encourage youth users to continue to practice skills after completing lessons). They then can use points as an in-app currency to buy colours, different accessories and backgrounds for their avatar character in the shop. We expect this will promote longer-term engagement in the app.  Data will be collected at two time points during the intervention delivery to account for the relatively long intervention course and in recognition that natural events in students’ lives (exam periods, holidays, etc.) may lead to variations in app engagement. The six and twelve month data collection will help us to understand factors influencing engagement over time and both immediate and longer term effects of the intervention.  Implementation Outcome Measures: We will use mixed methods to evaluate implementation outcomes. Quantitative indicators assessed will include uptake (number of youths enrolled in the study) and retention (number of enrolled youth who continue using the app across the 12-month period). To assess app acceptability, we will use an adapted version of the Acceptability of Health Apps Among Adolescents scale. We will also measure engagement with the app by examining metrics such as: frequency of logging in, and frequency of use of in-app activities.  Clinical Outcome Measures: In addition to capturing implementation metrics as described above, a suite of outcome measures will be used to assess health and well-being outcomes. The Warwick Edinburg Mental Wellbeing Scale (WEMWBS), a 14-item scale that uses positively worded statements to assess feeling and functioning aspects of mental well-being in the general population, will be the primary outcome measure. Other measures include: Depression Anxiety and Stress Scale (to measure symptoms and depression and anxiety), Brief Inventory of Thriving (to measure mental well-being), Connor-Davidson Resilience Scale (to measure resilience), Internet Addiction Test – short form (to measure internet addiction), Education Stress Scale for Adolescents (to measure stress related to educational pressure), and selected questions from the Vietnam Global Student Health Survey (to measure substance use, including tobacco products, alcohol, nitrous oxide and cannabis).  Additionally, we will conduct post-intervention focus group discussions (one in each school with 6-8 participants each at the twelve-month mark for each cohort) with study participants to explore their experiences of using the app. FGD questions will be co-designed with the V-YAC to ensure they capture appropriate themes that reflect factors influencing user satisfaction, engagement, and acceptability from the perspective of youth participants. This will allow us to: a) assess the appropriateness and acceptability of the app, and b) identify areas for refinement.  Understanding the perspectives of school staff is also essential to identify barriers and drivers to successful implementation. We will conduct semi-structured interviews with SHS, head teachers, and principals in each school (N=36 across all schools) to understand factors affecting implementation and potential scale-up. Interview questions will explore the attitudes of school staff related to the intervention (e.g. perceived impact on youth and school environment), perceived factors influencing implementation and scale-up (e.g. what would facilitate sustained implementation in their school, what resources would be needed for scale-up to other secondary schools).  1. Murphy J, Oanh PT, Goldsmith CH, Jones W, Nguyen VC. Introducing supported self-management for depression to primary care in Vietnam: A feasibility study in preparation for a randomized controlled trial. Fam Syst Health. 2018  2. Murphy, J.K., Xie, H., Nguyen, V.C. et al. Is supported self-management for depression effective for adults in community-based settings in Vietnam?: a modified stepped-wedge cluster randomized controlled trial. Int J Ment Health Syst 14, 8 (2020).  3. Paz Castro R, Haug S, Debelak R, Jakob R, Kowatsch T, Schaub MP. 2022. Engagement with a mobile phone-based life skills intervention for adolescents and its association with participant characteristics and outcomes: Tree-based analysis. J Med Internet Res. 24(1):e28638. \| \| ***5.2. Inclusion Criteria*** *Describe the participants being selected for this study, and list the criteria for their inclusion.* \| Criteria for participation will include: youth enrolled in grade 10 of participating secondary schools, who have access to a smartphone, who provide assent to participate and whose parents provide consent for their child to participate.  We will use a process of assent and parental consent, whereby youth participants sign a form to provide assent, and parents receive a form to sign to indicate whether or not they will allow their child to participate. While we originally planned to use a process of passive consent whereby parents only sign if they refuse to allow their child to participate, our partners at the Department of Education and Training in Hanoi, Vietnam requested that we use a standard consent process. We have therefore updated our protocol and parental consent forms accordingly to ensure we are compliant with local requirements. \| \| ***5.3. Exclusion Criteria*** *Include details if otherwise eligible participants will be excluded due to other characteristics. If no exclusion criteria are applicable, enter n/a.* \| Participants will be ineligible to participate if they are not in grade 10, not enrolled in participating secondary schools, they do not provide assent or if their parents do not provide informed consent. \| \| ***5.4. Recruitment*** *Provide a detailed description of the steps you will use to recruit participants. Include: a) How will prospective participants be identified?  b) By what means will recruitment be done (e.g., public posting, direct contact, third party recruitment, etc.)? c) Who will contact prospective participants? d) If recruitment will occur in person, what sites will be used (e.g. doctor's office, hospital clinic, etc.)? e) Attach all materials, including letters of initial contact, posters, scripts and advertisements, to Box 9.4.* \| We are collaborating with the Hanoi Department of Education and Training in Vietnam to help us identify secondary schools in three provinces in the northern region of Vietnam as our study sites. Selected study sites include two urban and two rural schools in the following provinces: Hanoi, Thai Binh and Hung Yen. Each province will enroll two cohorts in successive years with each cohort followed for one year. We will meet with the school staff (school health staff, head teachers, and school principals) to inform them about the study and the app-based intervention, and to engage two school staff from each school as study liaisons. To recruit students, PHAD study staff will make a presentation to Grade 10 students at all participating schools with details of the app and the study. Following the presentation, the study team will provide a QR code to a form where interested students can provide their name, their preferred method of communication (email and/or mobile phone number), and the email and mobile phone number of a parent or guardian. They will be required to provide the contact details of a parent or guardian in order to receive the assent form. Communicating directly with the study team will help to avoid any potential concerns or reluctance to make their teachers aware they are participating in the study.  Using the contact information provided by the students, the study team members will send a notice to parents by email and/or SMS explaining that their child is interested in participating in the study (see attached) with a link to the consent form. In addition, to ensure that parents do not ignore the communication from the study team or dismiss it as spam, the school will create a Zalo group for parents/ guardians of all Grade 10 students (this is a common way for schools to communicate with parents in Vietnam) that includes members of the PHAD study team informing them of the study, explaining that their child may have expressed interest in participating and that they should look for communication from the study team. Parents will also be able to use this Zalo group to ask any questions they have about the study and their child’s involvement. At the time this communication is sent out, a study-specific mobile phone number and email address will also be provided to parents so they are able to easily identify emails or SMS messages coming from the study team. Unless they have already sent in a signed consent form, parents will be sent two additional reminders from the study team, for a total of three communications. These will be sent in one-week intervals to allow sufficient time for the parent to read the consent form and respond. In addition to the contact information provided at the bottom of the consent form, the parent will also be invited to reply to the email or SMS message or to use the Zalo group to ask any questions about the study. \| \| ***5.5. Use of Records*** *If existing records (e.g., health records, course grade sheets or other records/databases) will be used to access information about potential participants, please describe how permission to access this information, and to collect and use this information, will be obtained.* \| N/A \| \| ***5.6. Summary of Procedures*** *Describe briefly in a step-by-step manner what the researcher will be doing with participants, after they have been recruited and consented.* \| Control and intervention cohorts will be enrolled in each school in successive years. The control cohort will be recruited first, and will be given the outcome assessments survey at baseline, 6 months, and 12 months marks, but will not receive access to the app. For ethical purposes, we will provide the control cohort access to the app after they have completed their final outcome assessment survey, but they will no longer provide outcome data and any in-app usage data (e.g. number and frequency of logins) which will be collected automatically by the app, will be excluded from the final analysis.  The intervention cohort, recruited the year after the control cohort, will have full access to the app and will complete the same outcome measures at baseline, 6 and 12 months. Their app-usage data will be included in the final analysis.  The outcome measures will be distributed via Qualtrics so students can complete them on their own devices. The suite of outcome measures (attached) consists of demographic information (to be completed only at baseline) and several validated scales including: the Positive Childhood Experiences scale, the Warwick Edinburgh Mental Wellbeing Scale, the Depression, Anxiety and Stress Scale- Youth Version, the Brief Inventory of Thriving, the Connor-Davison Resilience Scale- Vietnamese Version, the Internet Addiction Test-Short Version, the Educational Stress Scale for Adolescents, several questions related to alcohol, tobacco and substance use, experiences of bullying, peer influence and punishment that have been adapted from the Survey Assessment of Vietnamese Youth (SAVY), and questions about app acceptability (to be completed at months 6 and 12 only). All measures and the suite of outcome measures as a whole were reviewed by V-YAC members for face validity, clarity and appropriateness. On the advice of the V-YAC and due to the length of the questionnaire, we have added cartoons with encouraging messages throughout the survey to promote completion of the survey.  The app-based intervention (note: the app name is currently being finalized), co-designed with the Vietnamese Youth Advisory Council, aims to introduce life skills and self-management skills to promote general mental well-being and resilience among Vietnamese youth. With this app, youth users will have a fun and safe space to learn and practice six life skills areas (Problem-solving, Social Media and Well-being, Communication and Interpersonal Skills, Realistic Thinking, Coping with Emotions and Stress, and Goal Setting). Lessons (in the form of short texts) and activities (ex. multiple choice activities, blank worksheets, reflection questions) were adapted from evidence-based interventions developed in other contexts and were adapted in partnership with the V-YAC to ensure the skills and examples are culturally appropriate to the Vietnamese youth context. Youth users are free to explore the app at their own pace. Gamification features will also allow youth users to collect points in the app for completing each lesson, activity, or challenge (aimed to help encourage youth users to continue to practice skills after completing lessons). They then can use points to unlock and buy different accessories and backgrounds for their avatar (which is a gender neutral cartoon astronaut). Additionally, our app will include a mood check-in feature (where youth users can log and track their moods over time), guided breathing exercises, and a journaling feature. The app will collect the following data: 1) Demographic details at profile set up stage: name, school, city, gender, date of birth 2) Frequency of app log-in and duration of app engagement (how long are users using the app each time they open it) 4) Frequency and duration of feature engagement (how long are users interacting with each feature/lesson/activity)  Previous studies of youth engagement with app-based life skills interventions (1) show that receiving regular reminders by SMS or in-app notifications promotes engagement. Final details of the app’s function, including frequency and mode of delivery of reminders, will be confirmed during the co-design process (ongoing), but students will receive frequent reminders to engage with the intervention over the course of twelve months. We will collect data at baseline, 6 months, and 12 months for each cohort. Data will be collected at two time-points during the intervention delivery to account for the relatively long intervention course and in recognition that natural events in students’ lives (exam periods, holidays, etc.) may lead to variations in-app engagement. The six- and twelve-month data collection will help us to understand factors influencing engagement over time and both immediate and long-term effects of the intervention.  We will conduct outcome assessments via a Qualtrics online survey, which enrolled students may complete on their own devices or at school at each time point. This will ensure that outcome data collection is not dependent on app use. Some outcome measures (e.g. PHQ-8, GAD7 ) will also be included in the app for mental health self-assessment and to allow students to track their outcomes, but will not be collected as study data. We will also conduct focus group discussions with 6 – 8 participants at each school at the 12-month mark for each cohort to explore their experiences with the app, focusing on usability, appropriateness, and acceptability of app use.  We will distribute invitations to students inviting them to participate in a focus group using the contact information provided to the study team to invite their participation in the focus groups. We will aim to include students representing all genders and with different levels of engagement with the app in order to understand factors influencing engagement levels based on preliminary analysis of in-app data. We will generate lists of student participants according to gender and app use level (high, average and low) and will randomly select students from each group to receive invitations. We will repeat this process until we have recruited 6-8 from each school. The focus groups will take place in an empty classroom at school after school hours or on the weekend. The focus group will be led by a member of the PHAD study team.  We will conduct semi-structured interviews with SHS, head teachers, and principals in each school (N=36 across all schools) to understand factors affecting implementation and potential scale-up of this intervention. The interviews with school officials will take place on school premises (in offices or empty classrooms) after regular school hours or on the weekend. The interviews will be facilitated by a member of the PHAD study team. Interviews with school officials will be approximately one hour in duration.  (1) Paz Castro R, Haug S, Debelak R, Jakob R, Kowatsch T, Schaub MP. 2022. Engagement with a mobile phone-based life skills intervention for adolescents and its association with participant characteristics and outcomes: Tree-based analysis. J Med Internet Res. 24(1):e28638 \| \| ***5.7. Research Types*** *Select all that apply to your study. Please review the research methods descriptions before responding. If none apply, please select "None of these Methods"* \| Focus Groups \| \| **6. Participant Information and Consent Process - Behavioural Study**[[View Form]](https://rise.ubc.ca/rise/app/portal/smartform/edit?Project=com.webridge.entity.Entity%5BOID%5B100ACDEE22CB11EFFC91E27501565000%5D%5D&doValidation=False&Mode=smartform&WizardPageOID=com.webridge.entity.Entity%5BOID%5B723D0D1797B94343A736BD1D8E03ED89%5D%5D) \| \| \| ***6.1. Time to Participate*** \| Youth participation will consist of engaging in the app-based intervention at their own pace for one year and completing questionnaires at baseline, 6-month, and 12-month marks. The amount of time spent using the app will vary across the course of the study, but we will recommend that youth engage with the app for at least one hour a week initially (first three months), with the ability to return to the app to reinforce skills and concepts and to use features including daily mood tracking, journaling, etc. The majority of the students enrolled in the study will have no direct interaction with the study team and their participation will consist only of app use and completion of online outcome measures questionnaires at baseline, six and twelve months. A sub-population of students (6-8 students per school, total of n=24-32) will be invited to participate in post-intervention focus group discussions. These will be approximately 90 minutes in duration. The outcome measures questionnaires will take approximately 45 minutes to complete. Participants will be asked to complete this at enrolment (baseline), six and twelve months, for a total of 135 minutes during the year of study participation. We will also conduct semi-structured interviews with school staff (n=36), which will be approximately one hour in duration. \| \| ***6.2. Risks and Mitigation*** \| We anticipate that the risks associated with participation in the study will be low. The intervention takes a population-level approach to enhance life skills and self-management skills that are expected to be beneficial to all youth regardless of their mental health status, to promote mental well-being and prevent mental ill health. The PHQ-8 and GAD-7 measures will be included in the app so that students may use them to self-assess their mental well-being and to track changes over time. These scores will not be included in the overall dataset for the study. To ensure student confidentiality and privacy these scores will not be shared with anyone aside from the student when they complete the measure within the password protected app. While mental health services and supports are extremely limited in Vietnam, to promote safety students scoring 10 or above (suggesting moderate to severe depression or anxiety) on either measure will receive an in-app pop-up notification which states: “It looks like you might need some extra support! We recommend you talk to a trusted adult like a parent or guardian, teacher or school health staff member and visit a health centre or hospital. It’s ok to reach out for support when you need it! You can show them this scale to help you start the conversation about your well-being and the support you need.” This message will be repeated each time the study completes the measures and scores a 10 or above.  The intervention takes a strengths-based, skills-oriented approach to promote mental well-being and resilience among youth in Vietnam. The intervention combines principles from the WHO’s Life Skills Education for Children and Adolescents in Schools and the “Dealing with Depression: Antidepressant Skills for Teens”. The latter material is targeted at youth experiencing depression, however, the skills addressed in this guideline are relevant at the population level to promote well-being and prevent mental ill health. The app will consist of modules on the following topics: problem-solving and decision-making, communication and interpersonal skills, realistic thinking, stress reduction, goal setting and healthy use of social media. Where necessary, we adopted positive, strengths-based language that focuses on youth’s well-being rather than mental illnesses to minimize the stigma associated with mental health. The engagement of V-YAC throughout the adaptation of the intervention was essential to ensure the skills, exercises, and language written throughout the intervention were appropriate for youth.  All names and potentially identifying information collected through surveys and the app will be removed from all documentation, publications, etc. Study data, including transcripts and research notes, will only be available to the study team and will be securely stored and password-protected on UBC’s secure OneDrive platform. All data will be fully anonymized at the time of transcription and before analysis, publication, and dissemination. \| \| ***6.3. Potential Benefits*** \| The proposed Y-PRIME study will respond to a critical gap in mental health and well-being promotion and risk factor prevention for adolescents in Vietnam. Youth participants will have the benefit of learning skills that will promote their overall well-being and resilience when engaged in the app-based intervention. The intervention will include interactive skill-building modules that align the life and anti-depressant skills with exercises and activities tailored to respond to specific risk factors and concerns of Vietnamese youth. As identification and care for mental disorders in Vietnam is very low, the app will also include a self-assessment function, enabling youth to complete validated self-report measures for common mental disorders (e.g. GAD-7 for anxiety and PHQ-8 for depression). Youth will be able to see their own scores on these measures and track them over time. Youth scoring at or above the cut-off for moderate to severe depression or anxiety will receive an in-app pop-up encouraging them to talk to a trusted adult and to seek support from a health centre or hospital (see text in Box 6.2). This will help to ensure that students requiring formal mental health or emergency support are identified and referred to appropriate care as is possible within Vietnam's limited mental health system. . \| \| ***6.4. Impacts on Community*** \| The engagement of key stakeholders including policymakers, school staff, and youth leaders will promote successful implementation and will help to lay the groundwork for scale-up throughout the country. The Government of Vietnam has demonstrated an increased interest and commitment to the use of digital technologies to deliver mental health care, a commitment that has grown during the COVID-19 pandemic. Project partners the Hanoi Department of Education, the Vietnamese Ministry of Labour, Invalids [sic], and Social Affairs, and the Hanoi Youth Union have all identified children and youth mental health as priorities and are engaged in this project. The integrated knowledge translation approach taken throughout the study will promote long term engagement to support scale-up of the intervention. The engagement of our Vietnam Youth Advisory Council will help to ensure the app is appropriate and responsive to the needs and context of youth in Vietnam, helping to promote uptake and sustained engagement with the app. Finally, mental health promotion, prevention and care resources are extremely limited in Vietnam. This app will help to provide a much-needed resource for youth at a population level that has the potential to support improved mental well-being and resilience in the long term. \| \| ***6.5. Reimbursement and Incentives*** \| Participants will be entered into a lucky draw (per cohort) for gift cards prized at 500,000 VND (approximately CAD 27.00). The draw for each cohort will take place at the end of the 12 month study period in which they are participating. All participants who enroll in the study, regardless of whether they choose to withdraw, will be eligible for the lucky draw.  Participating schools will assign two staff members per school as study liaisons. These staff members will be provided with a stipend of VND 1 million (approximately CAD $50) per month.  School staff participating in post-intervention interviews will be provided with an honorarium of VND 200,00 (approximately CAD $10.00), which is customary for research participation in Vietnam. \| \| ***6.6. Obtaining Consent*** *Include details of where and when consent will be obtained and how it will be documented.* \| Study staff will make a presentation to Grade 10 students at all participating schools with details of the app and the study. Following the presentation, the study team will provide a QR code to a form where interested students can provide their name, their preferred method of communication (email and/or mobile phone number), and the email and mobile phone number of a parent or guardian. They will be required to provide the contact details of a parent or guardian in order to receive the form to indicate their assent. Communicating directly with the study team will help to avoid any potential concerns or reluctance to make their teachers aware they are participating in the study.  Using the contact information provided by the students, the study team members will send a notice to parents by email and/or SMS explaining that their child is interested in participating in the study with a link to the consent form. In addition, to ensure that parents do not ignore the communication from the study team or dismiss it as spam, the school will create a Zalo group for parents/ guardians of all Grade 10 students. It is standard practice for schools to use Zalo, a Vietnamese social media platform that is similar to WhatsApp, to communicate with parents/ guardians. Parents will not be provided with the consent form on the group chat. They will be sent the consent form directly by the study team by email and/ SMS. Zalo will be used to inform parents about the study in general, to ask them to look for communications from the study team in their email inbox or on their phone. In addition to directly contacting the study team by email, phone or SMS, the Zalo group will allow parents to ask any questions about the study to a PHAD team member who will be included in the Zalo group.  Given the age of the parents (likely 30s-50s) and the very high levels of Smartphone use in Vietnam, we anticipate that they will have access to a mobile phone and Internet connection to allow them to access the consent form. In the event that they are unable or have a preference not to complete the form online, a study team member will review the consent form over the phone and take verbal details. Details of parents' consent will therefore be collected via the Qualtrics form or verbally over the phone by a member of the Vietnam study team. \| \| ***6.6.A. Waiver of Consent*** \| N/A \| \| ***6.7. Time to Decide*** \| Participants will be informed about the study and invited to participate one month before enrollment, giving students and their parents/ guardians sufficient time to review the study details, ask any questions of the study team and/or school staff and provide consent if they wish to participate. We will send reminders using the same channels used to inform them about the study (school announcements, social media, assemblies, flyers from school health staff) two weeks and then three days prior to the enrollment deadline. \| \| ***6.8. Capacity to Consent*** *Will participants have the capacity to give fully informed consent on their own behalf?* \| No \| \| ***6.8.A.****Provide details of the nature of the incapacity (for instance, young age, mental or physical condition).* \| Study participants will be 15 years old and will therefore have the option to provide assent, while their parents will provide consent, whereby they sign a consent form to indicate whether or not they allow their child to participate in the study. \| \| ***6.8.B.****If a participant does not have the capacity to give fully informed consent, who will consent on their behalf? Ensure the relevant consent form (parent/caregiver, substitute decision maker, legally authorized representative) is attached to page 9.* \| Parents will provide consent by signing a consent form to indicate whether or not they agree to allow their child to participate in the study. \| \| ***6.8.C.****If a participant does not have the capacity to give fully informed consent, will they be able to give assent to participate?* \| yes \| \| ***6.8.D.****If yes, explain how assent will be sought.  Please be sure to attach copies of the assent form to page 9.* \| Youth indicating their interest in the study will be provided with an assent form (attached) providing details of the study, what their voluntary involvement will entail. Students will be asked to provide assent by signing this form in order to participate in the study. \| \| ***6.9. Ongoing Consent*** \| Participants will be asked to provide informed assent for their one-year participation in the study at enrollment. This assent, along with their parents consent, will remain active for the duration of the study. Participants will be informed during the assent process that they are able to leave the study at any time with no negative consequences. \| \| ***6.10. Provisions for Consent (e.g., special assistance, Braille, translations/translator)*** \| Written, online assent forms will be provided to youth participants in Vietnamese. English and Vietnamese versions of the assent and consent form are currently attached. \| \| ***6.11. Restrictions on Disclosure*** \| N/A \| \| **7. Number of Participants - Behavioural Study**[[View Form]](https://rise.ubc.ca/rise/app/portal/smartform/edit?Project=com.webridge.entity.Entity%5BOID%5B100ACDEE22CB11EFFC91E27501565000%5D%5D&doValidation=False&Mode=smartform&WizardPageOID=com.webridge.entity.Entity%5BOID%5B08C2024131346D4EAD6888D00400FDB1%5D%5D) \| \| \| ***7.1. External Approvals***  ***A.****Other Institutions:* \| yes \| \| ***B.****Please select "Add" to enter the name of the institution and attach the approval letter if received.* \| \| **Name of Institution** \|  \| \| --- \| --- \| \| Institute of Population, Health and Development \|  \| \|  \|  \| \| \| ***C.****Other Jurisdiction or Country (if "NO," go to 7.1.G):* \| yes \| \| ***D.****Please select "Add" to enter the name of the jurisdiction or country and if you have already received approval attach the approval letter.* \| \| **Name of Jurisdiction or Country** \|  \| \| --- \| --- \| \| Vietnam \|  \| \|  \|  \| \| \| ***E.****Has a Request for Ethics Approval been submitted to the institution or responsible authority in the other jurisdiction or country? (****Append a copy of any such document to this application once it is received****).* \| no \| \| ***F.****If a Request for Approval has****not been****submitted, provide the reasons below:* \| Our partner institution, the Institute of Population, Health and Development (PHAD), will seek approval from its Institutional Review Board. This process cannot be undertaken until ethics approval has been obtained from UBC. The approval certificate from PHAD’s IRB will be added to this application following approval. \| \| ***G.****Does this research focus on Indigenous peoples, communities or organizations?* \| no \| \| ***G.1.A.****Will the research be conducted on Indigenous reserves, Métis settlement(s), or lands governed under a self-government agreement or an Inuit or First Nations land claims agreement?* \|  \| \| *If yes, please provide details:* \|  \| \| ***G.1.B.****Do any of the criteria for participation include membership in an Indigenous community, group of communities, or organization, including urban Indigenous populations?* \|  \| \| *If yes, please provide details:* \|  \| \| ***G.1.C.****Does the research seek input from participants regarding a community’s cultural heritage, artifacts, traditional knowledge or unique characteristics?* \|  \| \| *If yes, please provide details:* \|  \| \| ***G.1.D.****Will Indigenous identity or membership in an Indigenous community be used as a variable for the purposes of analysis?* \|  \| \| *If yes, please provide details:* \|  \| \| ***G.1.E.****Will the results of the research refer to Indigenous communities, peoples, language, history or culture?* \|  \| \| *If yes, please provide details:* \|  \| \| ***G.2. Community Engagement***  ***G.2.A.****If you answered yes to questions a), b), c), d), or e), have you initiated or do you intend to initiate an engagement process with the Indigenous collective, community or communities for this study?* \|  \| \| ***G.2.B.****If you answered "Yes" to question G.2.A., describe the process that you have followed or will follow with respect to community engagement. Include the role or position of those consulted, including their names if appropriate. Attach any documentation of consultations (i.e. formal research agreement, letter of approval, email communications, etc.) below.* \|  \| \| *Attachment:* \|  \| \| ***G.3. No community consultation or engagement*** *If you answered "no" to question G.2.A., briefly describe why community engagement will not be sought and how you can conduct a study that respects Indigenous communities and participants in the absence of community engagement.* \|  \| \| ***H.****Registration for Publication of Clinical Trials.* \| no \| \| *If 'Yes', click 'Add' to enter the following information.* \| \| **Has it been registered?** \| **Indicate the Authorized Registry used:** \| **Enter your Clinical Trial unique identifier:** \|  \| \| --- \| --- \| --- \| --- \| \| \| ***7.2. Number of Participants   A.****How many participants will take part in the entire study (i.e., world-wide)?* \| 1800 \| \| ***B.****How many participants will take part at institutions covered by this Research Ethics Approval?* \| 1800 \| \| ***7.3. Principal Investigator and Research Team Experience*** \| The research team consists of a multidisciplinary team of experts in global mental health, digital mental health, implementation science, youth mental health and patient-engaged research including researchers at all career stages. PI Dr. Lam is Professor and BC Leadership Chair in Depression Research, Associate Head for Graduate Education of the Department of Psychiatry at UBC and the past Executive Chair of the Canadian Network for Mood and Anxiety Treatments (CANMAT), a recognized provider of high-integrity clinical guidelines and knowledge translation (KT). He has extensive experience in digital health research including as co-PI of the GACD-funded EMBED Canada-China study. Co-PA Dr. Nguyen is Director of PHAD in Hanoi, Vietnam. He has been the co-PI of several mental health studies in Vietnam and has extensive experience in research and policy engagement in the country. He will provide leadership and oversight for the research team in Vietnam. Co-PIs include Dr. Murphy, a Research Associate in the Department of Psychiatry at UBC and the Executive Director of the APEC Digital Hub for Mental Health (mentalhealth.apec.org). She has worked in Vietnam since 2014, including for her PhD research, and has been a co-investigator on several studies in the country. She has expertise in implementation science, conducting mixed methods studies and randomized controlled trials and will participate in stakeholder engagement activities, data collection, and analysis. Dr. O’Neil, Professor in the Faculty of Health Sciences (FHS) at Simon Fraser University, is the NPA on several studies in Vietnam and has extensive experience in global health. Dr. Minas is Associate Professor and Head of the Global and Cultural Health Unit at the University of Melbourne. He has worked extensively in mental health systems and policy research in Vietnam. Dr. Michalak is a Professor in the Department of Psychiatry at UBC and recipient of the 2018 CIHR Gold Leaf Prize for Transformation in Patient Engagement. Her expertise is in patient-engaged research, including in co-design of digital mental health interventions. Dr. Hayashi is an Assistant Professor in FHS at SFU and St. Paul’s Hospital Chair in Substance Use Research. She has expertise in epidemiology and community-based research, including in substance-use research in Asia. Dr. Barbic is an Assistant Professor in Occupational Therapy at UBC and is the Research Lead at Foundry BC. She has expertise in youth-engaged, strengths-based mental health and substance use research. Dr. Samji is an Assistant Professor in FHS at SFU and is a Senior Scientist at the BC Centers for Disease Control. She is an epidemiologist with expertise in youth mental health and wellbeing. Ms. Chau is a PhD candidate in FHS at SFU and has worked in mental health research in Vietnam for several years. \| \| **8. Confidentiality - Behavioural Study**[[View Form]](https://rise.ubc.ca/rise/app/portal/smartform/edit?Project=com.webridge.entity.Entity%5BOID%5B100ACDEE22CB11EFFC91E27501565000%5D%5D&doValidation=False&Mode=smartform&WizardPageOID=com.webridge.entity.Entity%5BOID%5B7B4AD89875570248ADAA8704E1A9BCF5%5D%5D) \| \| \| ***8.1. Security of Data During the Course of the Study*** \| All data collected via the mobile app (demographic profile details, frequency and duration of app log-in and engagement, frequency and duration of feature/lesson/activity engagement) will be stored in a secure folder on PHAD’s server at the PHAD’s office. Though app users will be able to use the in-app PHQ-8 and GAD-7 scales for self-assessment and monitoring, data will not be included in the study dataset final analysis. PHQ-8 and GAD-7 scores will be retained within the app for self-monitoring purposes and will be subject to the same secure storage as other in-app data. Recorded audio files and MSWord transcripts from focus group discussions with youth and semi-structured interviews with school staff will be immediately uploaded and saved on UBC’s OneDrive secure file-sharing server and will be password protected. The outcome measures data will be collected via UBC’s Survey Tool (Qualtrics). Information collected using the Survey Tool is kept secure using measures including data encryption. Data will also be uploaded onto UBC’s OneDrive and will be password protected. Only members of the study team who are directly engaged in the transcription or analysis of data files will have access to the UBC’s OneDrive folder and PHAD’s secure folder. \| \| ***8.2. Access to Data*** \| Only members of the study team from UBC and PHAD will have access to the data. These include PI's Dr. Lam and Dr. Nguyen, co-investigator Dr. Murphy. Research assistants and graduate students at PHAD and UBC will also have access to the data and will participate in transcription and analysis. All staff who access the data will be informed about standard procedures related to de-identification of data and for maintaining the privacy and confidentiality of research participants. \| \| ***8.3. Protection of Personal Information*** \| After the full dataset is complete, each record will be assigned a unique code. The dataset will then be divided into two parts: one containing only identifiable information (such as names and school) and another containing the remaining data for analysis. Both parts will include the unique code for each record, allowing us to cross-reference them if necessary. The dataset without identifiable information will be used for analysis, while the file containing identifiable data will be stored in a secure, password-protected and encrypted folder on PHAD’s server. Access to this file will be strictly limited to members of the study team. All potentially identifying information (e.g. names or initials, location of residence, specific occupation or school, etc.) will be excluded from study datasets at the time of transcription. No identifying features will be attributed to study participants in any resulting publications, presentations or other knowledge dissemination activities. In transcripts and when making use of illustrative quotations in the reporting of study findings, participants will be identified with a randomly assigned number (e.g. P1, P2) which will not be associated with the participants' identity. To register for app use, we will collect participants’ name, email, phone number, and school name. The raw data collected through the app will include this information, however, the identifying information will be removed during analysis. The app data will be stored on PHAD’s secure server and backed up weekly. PHAD will assign one researcher the responsibility of removing identifying information from the study datasets. \| \| ***8.4. Transfer of Data*** *Will any data be transferred (made available) to persons or agencies outside the lead University or Health Authority?* \| yes \| \| *If yes, describe in detail what information will be released, to whom, how the data will be transferred, how and where it will be stored and what safeguards will be used to protect the identity of participants and the privacy of their data. Attach the data transfer agreement if applicable.* \| Data will be collected in Vietnam by UBC and PHAD researchers. PHAD partners will be provided with access to a secure folder on UBC's OneDrive platform, and will be instructed to share files via uploading to this platform instead of sharing any data by e-mail or other file-sharing options. Any data shared with PHAD by UBC will be similarly uploaded directly to the secure One Drive folder. \| \| ***8.5. Retention and Destruction of Data*** \| All data files, including Word documents, datasets in Excel and audio files, will be saved in UBC's OneDrive platform for seven years, after which they will be fully deleted. The Principal Investigator Dr. Raymond Lam will be responsible for all study data. All app data will be stored in PHAD’s secure server for five years, after which they will be fully deleted. \| \| ***8.6. Future Use of Data*** \| All data files, including Word documents, datasets in Excel and audio files, will be saved in UBC's OneDrive platform for seven years, after which they will be fully deleted. All app data will be stored in PHAD’s secure server for five years, after which they will be fully deleted. Data collected in this study might be used in future studies and analyses. \| \| ***8.7. Feedback to Participants*** *Please provide information regarding your plans for communicating study results to participants. See the guidance notes for more information and respond to the bullet points as needed.* \| In addition to academic outputs (e.g. peer-reviewed publications, conference presentations) we will communicate study results with government partners via presentations at in-person meetings and a policy brief. We will also prepare an infographic which will be distributed via a flyer and on social media to participating schools in order to inform students, parents and school staff about the results of the study. \| \| **9. Documentation - Behavioural Study**[[View Form]](https://rise.ubc.ca/rise/app/portal/smartform/edit?Project=com.webridge.entity.Entity%5BOID%5B100ACDEE22CB11EFFC91E27501565000%5D%5D&doValidation=False&Mode=smartform&WizardPageOID=com.webridge.entity.Entity%5BOID%5B466ADFB01D80C044BDD0927AB56FCA15%5D%5D) \| \| \| ***9.1. Research Proposal*** \| \| **Document Name** \| **Version** \| **Date** \| **Password (if applicable)** \|  \| \| --- \| --- \| --- \| --- \| --- \| \| Y-PRIME Research Proposal \|  \| Monday, May 30, 2022 \|  \| [[View]](https://rise.ubc.ca/rise/sd/Doc/0/KET7SBQ2R08UVVSHS9QG2LIG00/Lam%20et%20al_GACD%20Y-PRIME%20Proposal_FINAL.pdf) \| \|  \|  \|  \|  \|  \| \| \| ***9.2. Documentation of Consent*** \| \| **Document Name** \| **Version** \| **Date** \| **Password (if applicable)** \|  \| \| --- \| --- \| --- \| --- \| --- \| \| [ENG] Parents Phase 2 Consent Form - Intervention \| 3 \| Wednesday, October 16, 2024 \|  \| [[View]](https://rise.ubc.ca/rise/sd/Doc/0/64I8DNCBSS8UU1CIS9QG2LIG00/%5BENG%5D%20Parents%20Phase%202%20Consent%20Form%20-%20Intervention%20V3.docx) \| \|  \|  \|  \|  \|  \| \| [ENG] Parents Phase 2 Consent Form - Control \| 1 \| Wednesday, October 16, 2024 \|  \| [[View]](https://rise.ubc.ca/rise/sd/Doc/0/94Q33VCBSS8UU1CIS9QG2LIG00/%5BENG%5D%20Parents%20Phase%202%20Consent%20Form%20-%20Control%20V1.docx) \| \|  \|  \|  \|  \|  \| \| [VN] Parents Phase 2 Consent Form - Intervention \| 2 \| Wednesday, October 16, 2024 \|  \| [[View]](https://rise.ubc.ca/rise/sd/Doc/0/C4KFIFSBSS8UU1CIS9QG2LIG00/%5BVN%5D%20Parents%20Phase%202%20Consent%20Form%20-%20Intervention%20V2.docx) \| \|  \|  \|  \|  \|  \| \| [VN] Parents Phase 2 Consent Form - Control \| 1 \| Wednesday, October 16, 2024 \|  \| [[View]](https://rise.ubc.ca/rise/sd/Doc/0/DKDDCJKBSS8UU1CIS9QG2LIG00/%5BVN%5D%20Parents%20Phase%202%20Assent%20Form-%20Control%20V1.docx) \| \|  \|  \|  \|  \|  \| \| [VN] School Staff Interview Consent Form - V2 \| 2 \| Tuesday, September 10, 2024 \|  \| [[View]](https://rise.ubc.ca/rise/sd/Doc/0/EEGRC7JH6G8UU14IS9QG2LIG00/%5BVN%5D%20School%20Staff%20Interview%20Consent%20Form%20-%20V2.docx) \| \|  \|  \|  \|  \|  \| \| [ENG] School Staff Interview Consent Form - V2 \| 2 \| Tuesday, September 10, 2024 \|  \| [[View]](https://rise.ubc.ca/rise/sd/Doc/0/C6SE2VRH6G8UU14IS9QG2LIG00/%5BENG%5D%20School%20Staff%20Interview%20Consent%20Form%20-%20V2.docx) \| \|  \|  \|  \|  \|  \| \| \| ***9.3.  Documentation of Assent*** \| \| **Document Name** \| **Version** \| **Date** \| **Password (if applicable)** \|  \| \| --- \| --- \| --- \| --- \| --- \| \| [ENG] Youth Participants Phase 2 Consent Form - Intervention \| 3 \| Wednesday, October 16, 2024 \|  \| [[View]](https://rise.ubc.ca/rise/sd/Doc/0/HC0P2T4BSS8UU1CIS9QG2LIG00/%5BENG%5D%20Youth%20Participants%20Phase%202%20Assent%20Form%20-%20Intervention%20V3.docx) \| \|  \|  \|  \|  \|  \| \| [VN] Youth Participants Phase 2 Consent Form - Intervention \| 3 \| Wednesday, October 16, 2024 \|  \| [[View]](https://rise.ubc.ca/rise/sd/Doc/0/LT5QTGSBSS8UU1CIS9QG2LIG00/%5BVN%5D%20Youth%20Participants%20Phase%202%20Assent%20Form%20-%20Intervention%20V3.docx) \| \|  \|  \|  \|  \|  \| \| [VN] Youth Participants Phase 2 Consent Form - Control \| 1 \| Wednesday, October 16, 2024 \|  \| [[View]](https://rise.ubc.ca/rise/sd/Doc/0/NCU57GCBSS8UU1CIS9QG2LIG00/%5BVN%5D%20Youth%20Participants%20Phase%202%20Assent%20Form%20-%20Control%20V1.docx) \| \|  \|  \|  \|  \|  \| \| [ENG] Youth Participants Phase 2 Assent Form - Control \| 1 \| Wednesday, October 9, 2024 \|  \| [[View]](https://rise.ubc.ca/rise/sd/Doc/0/AQ3DAGSM488UU1KIS9QG2LIG00/%5BENG%5D%20Youth%20Participants%20Phase%202%20Assent%20Form%20-%20Control%20V1.docx) \| \|  \|  \|  \|  \|  \| \| \| ***9.4. Advertisement to Recruit Participants****(Ads, Posters, letters of initial contact, etc).* \| \| **Document Name** \| **Version** \| **Date** \| **Password (if applicable)** \|  \| \| --- \| --- \| --- \| --- \| --- \| \| [ENG] Recruitment Flyer - YPRIME Phase 2 \| 2 \| Tuesday, September 10, 2024 \|  \| [[View]](https://rise.ubc.ca/rise/sd/Doc/0/U6B0R93H6G8UU14IS9QG2LIG00/%5BENG%5D%20Recruitment%20Flyer%20-%20YPRIME%20Phase%202%20-%20V2.docx) \| \|  \|  \|  \|  \|  \| \| [VN] Recruitment Flyer - YPRIME Phase 2 \| 2 \| Tuesday, September 10, 2024 \|  \| [[View]](https://rise.ubc.ca/rise/sd/Doc/0/VMMEHBJH6G8UU14IS9QG2LIG00/%5BVN%5D%20Recruitment%20Flyer%20-%20YPRIME%20Phase%202%20-%20V2.docx) \| \|  \|  \|  \|  \|  \| \| \| ***9.5. Questionnaire, Questionnaire Consent Cover Letter, Tests, Interview Scripts, etc.*** \| \| **Document Name** \| **Version** \| **Date** \| **Password (if applicable)** \|  \| \| --- \| --- \| --- \| --- \| --- \| \| [ENG] Y-PRIME Outcome Measures \| 3 \| Monday, October 7, 2024 \|  \| [[View]](https://rise.ubc.ca/rise/sd/Doc/0/4D7J91K4VO8UU1CIS9QG2LIG00/%5BENG%5D%20Y-PRIME%20Outcome%20Measures_V3.docx) \| \|  \|  \|  \|  \|  \| \| [VN] Y-PRIME Outcome Measures \| 3 \| Monday, October 7, 2024 \|  \| [[View]](https://rise.ubc.ca/rise/sd/Doc/0/5AB3T344VO8UU1CIS9QG2LIG00/%5BVN%5D%20Y-PRIME%20Outcome%20Measures_V3.docx) \| \|  \|  \|  \|  \|  \| \| [ENG] Youth Participants Focus Group Questions \|  \| Wednesday, June 26, 2024 \|  \| [[View]](https://rise.ubc.ca/rise/sd/Doc/0/EQ4VRDQ3KS8UVVSHS9QG2LIG00/%5BENG%5D%20Youth%20Participants%20Focus%20Group%20Discussion%20Questions.docx) \| \|  \|  \|  \|  \|  \| \| [VN] Youth Participants Focus Group Questions \|  \| Wednesday, June 26, 2024 \|  \| [[View]](https://rise.ubc.ca/rise/sd/Doc/0/JCIVUL23KS8UVVSHS9QG2LIG00/%5BVN%5D%20Youth%20Participants%20Focus%20Group%20Discussion%20Questions.docx) \| \|  \|  \|  \|  \|  \| \| [ENG] School Staff Interview Questions \|  \| Wednesday, June 26, 2024 \|  \| [[View]](https://rise.ubc.ca/rise/sd/Doc/0/LL9P9QI3KS8UVVSHS9QG2LIG00/%5BENG%5D%20School%20Staff%20Interview%20Questions.docx) \| \|  \|  \|  \|  \|  \| \| [VN] School Staff Interview Questions \|  \| Wednesday, June 26, 2024 \|  \| [[View]](https://rise.ubc.ca/rise/sd/Doc/0/NT0QVFA3KS8UVVSHS9QG2LIG00/%5BVN%5D%20School%20Staff%20Interview%20Questions.docx) \| \|  \|  \|  \|  \|  \| \| \| ***9.6. Letter of Initial Contact*** \| \| **Document Name** \| **Version** \| **Date** \| **Password (if applicable)** \|  \| \| --- \| --- \| --- \| --- \| --- \| \| [ENG] Email_SMS to parents about consent form link \| 2 \| Wednesday, October 16, 2024 \|  \| [[View]](https://rise.ubc.ca/rise/sd/Doc/0/RSMHC3SBSS8UU1CIS9QG2LIG00/%5BENG%5D%20Email_SMS%20to%20parents%20about%20consent%20form%20link%20V2.docx) \| \|  \|  \|  \|  \|  \| \| [VN] Email_SMS to parents about consent form link \| 2 \| Wednesday, October 16, 2024 \|  \| [[View]](https://rise.ubc.ca/rise/sd/Doc/0/TCENVGKBSS8UU1CIS9QG2LIG00/%5BVN%5D%20Email_SMS%20to%20parents%20about%20consent%20form%20V2.docx) \| \|  \|  \|  \|  \|  \| \| \| ***9.7. Other Documents*** \| \| **Document Name** \| **Version** \| **Date** \| **Password (if applicable)** \|  \| \| --- \| --- \| --- \| --- \| --- \| \| Y-PRIME App Specification Template \|  \| Wednesday, October 16, 2024 \|  \| [[View]](https://rise.ubc.ca/rise/sd/Doc/0/VCFKAKSCNC8UU1CIS9QG2LIG00/Y-PRIME%20Application%20Specification%20Template.docx) \| \|  \|  \|  \|  \|  \| \| Screenshots of app \|  \| Tuesday, September 10, 2024 \|  \| [[View]](https://rise.ubc.ca/rise/sd/Doc/0/8TJ32QJHF88UU14IS9QG2LIG00/Screenshots%20of%20app.docx) \| \|  \|  \|  \|  \|  \| \| [ENG] Intervention Domain 1 - Problem-solving \|  \| Tuesday, September 10, 2024 \|  \| [[View]](https://rise.ubc.ca/rise/sd/Doc/0/STE9GA3HF48UU14IS9QG2LIG00/Intervention%20Domain%201%20-%20Problem-solving%20and%20Decision%20Making.docx) \| \|  \|  \|  \|  \|  \| \| Safe Research Plan \|  \| Friday, September 6, 2024 \|  \| [[View]](https://rise.ubc.ca/rise/sd/Doc/0/VR613GBH6O8UU14IS9QG2LIG00/Lam%20signed%2C%20Safe%20Research%20Plan.docx) \| \|  \|  \|  \|  \|  \| \| \| ***9.8. Websites and Social Media*** \|  \| \| **10. Fee for Service - Behavioural Study**[[View Form]](https://rise.ubc.ca/rise/app/portal/smartform/edit?Project=com.webridge.entity.Entity%5BOID%5B100ACDEE22CB11EFFC91E27501565000%5D%5D&doValidation=False&Mode=smartform&WizardPageOID=com.webridge.entity.Entity%5BOID%5BE2B93BDF4F5FBE488CCB1553CA8358E8%5D%5D) \| \| \| ***How to submit*** *Please indicate which of the following methods of payment will be used for this application:* \| N/A (Not funded by an Industry For-Profit sponsor) \| \| ***Please wait for the invoice from the UBC Behavioural Research Ethics Board (BREB) to submit payment. The invoice will detail payment instructions and wire transfer information.***  ***Contact information regarding where to send the invoice.*** \|  \| \| **11. Hospital Information - Providence Health Care**[[View Form]](https://rise.ubc.ca/rise/app/portal/smartform/edit?Project=com.webridge.entity.Entity%5BOID%5B100ACDEE22CB11EFFC91E27501565000%5D%5D&doValidation=False&Mode=smartform&WizardPageOID=com.webridge.entity.Entity%5BOID%5BFFC18C4ECC32C842BBE2E97EB82F2D8A%5D%5D) \| \| \| ***11.1.  11.1.A.*** *Which of the following hospital services are required for the conduct of your research? (Please check all that apply).* \| N/A \| \| ***11.1.B.*** *If "Other" provide details below.* \|  \| \| ***11.2.  11.2.A.*** *Which of the following hospital areas will be required to provide services for the conduct of the research? If the PI for the research is employed by the hospital area in question and has obtained approval for use of his or her own area, please do not select the relevant option. (Please check all that apply).* \| N/A \| \| ***11.2.B.*** *Provide details below of other hospital areas affected by the study.* \|  \| \| ***11.3.*** *Does the Principal Investigator in Box 1.1 have a UBC appointment?* \| yes \| \| *Declaration Form if PI doesn't have UBC appointment* \| [[View]](https://rise.ubc.ca/rise/sd/CustomLayouts/PrintSmartForms?Project=com.webridge.entity.Entity%5bOID%5b100ACDEE22CB11EFFC91E27501565000%5d%5d) \| \| **11. Hospital Information - Vancouver Coastal Health**[[View Form]](https://rise.ubc.ca/rise/app/portal/smartform/edit?Project=com.webridge.entity.Entity%5BOID%5B100ACDEE22CB11EFFC91E27501565000%5D%5D&doValidation=False&Mode=smartform&WizardPageOID=com.webridge.entity.Entity%5BOID%5BDBA0F28ED72F3F47B84C956527C405B9%5D%5D) \| \| \| ***11.1.A.*** *Have you already received approval from VCHA to conduct this study?* \| no \| \| ***11.1.B.*** *If Yes, please provide the VCHA/VCHRI approval number (e.g. V06-0000)* \|  \| \| ***11.2.A.*** *Does the Principal Investigator in question 1.1 have a UBC appointment?* \| yes \| \| ***11.2.B.*** *If No, please attach the declaration form.* \| [[View]](https://rise.ubc.ca/rise/sd/CustomLayouts/PrintSmartForms?Project=com.webridge.entity.Entity%5bOID%5b100ACDEE22CB11EFFC91E27501565000%5d%5d) \| \| ***11.3.*** *If your research study involves Vancouver Community sites, have you consulted with the VCHRI Research Facilitator?* \| no \| \| **12. Save Application - Human Ethics**[[View Form]](https://rise.ubc.ca/rise/app/portal/smartform/edit?Project=com.webridge.entity.Entity%5BOID%5B100ACDEE22CB11EFFC91E27501565000%5D%5D&doValidation=False&Mode=smartform&WizardPageOID=com.webridge.entity.Entity%5BOID%5BA99751155DE6B14A90C16B78291BA9CB%5D%5D) \| \| |
| \|  \|  \|  \| \| --- \| --- \| --- \| |
